# Building Block-Based Binding Predictions for DNA-Encoded Libraries

**DOI:** 10.1021/acs.jcim.3c00588

**Published:** 2023-08-14

**Authors:** Chris Zhang, Mary Pitman, Anjali Dixit, Sumudu Leelananda, Henri Palacci, Meghan Lawler, Svetlana Belyanskaya, LaShadric Grady, Joe Franklin, Nicolas Tilmans, David L. Mobley

**Affiliations:** † Department of Chemistry, University of California, Irvine, 1120 Natural Sciences II, Irvine, California 92697, United States; ‡ Department of Pharmaceutical Sciences, University of California, Irvine, 856 Health Sciences Road, Irvine, California 92697, United States; ¶ Anagenex, 20 Maguire Road Suite 302, Lexington, Massachusetts 02421, United States

## Abstract

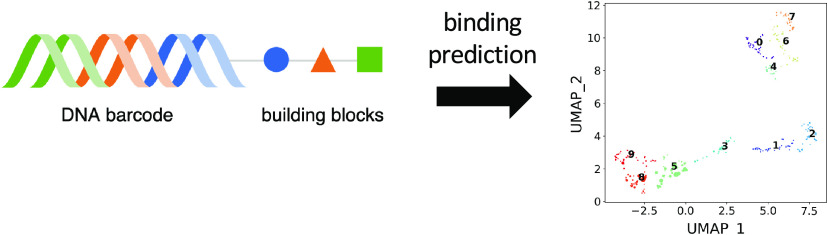

DNA-encoded libraries (DELs) provide the means to make and screen millions of diverse compounds against a target of interest in a single experiment. However, despite producing large volumes of binding data at a relatively low cost, the DEL selection process is susceptible to noise, necessitating computational follow-up to increase signal-to-noise ratios. In this work, we present a set of informatics tools to employ data from prior DEL screen(s) to gain information about which building blocks are most likely to be productive when designing new DELs for the same target. We demonstrate that similar building blocks have similar probabilities of forming compounds that bind. We then build a model from the inference that the combined behavior of individual building blocks is predictive of whether an overall compound binds. We illustrate our approach on a set of three-cycle OpenDEL libraries screened against soluble epoxide hydrolase (sEH) and report performance of more than an order of magnitude greater than random guessing on a holdout set, demonstrating that our model can serve as a baseline for comparison against other machine learning models on DEL data. Lastly, we provide a discussion on how we believe this informatics workflow could be applied to benefit researchers in their specific DEL campaigns.

## Introduction

Drug discovery campaigns have increasingly adopted DNA-encoded libraries (DELs) in recent years because they allow for relatively cheap and rapid exploration of diverse areas of chemical space.^1−6^ In DELs, a concept first introduced by Brenner and Lerner,^7^ scientists sequentially couple small molecules known as building blocks via split-and-pool combinatorial synthesis. The process tags each building block with a unique DNA oligomer such that each final library member is covalently attached to a record of its synthesis in the form of a sequenceable DNA barcode. Researchers then incubate the entire library with a target of interest and wash away any compounds that do not bind. Finally, experimentalists amplify and sequence the DNA barcodes of the observed binders and further investigate any compounds with detected DNA read counts as potential binders.^8,9^ Typically, DELs incorporate two to four cycles of encoding and chemistry, which can achieve a diversity of up to billions of unique compounds.^10−12^

Given the large combinatorial scale of DELs, selection data can be quite noisy due to issues such as variable reaction yields and formation of truncates,^13−15^ as well as errors within experimental procedures and noise during DNA sequencing.^16,17^ These sources of noise have made it common to analyze selection data with computational models to prevent wasting time and resources resynthesizing and evaluating unproductive candidates. Recent work suggests how machine learning approaches can denoise DEL data^13,15,18^ and identify promising candidates in out-of-sample data.^19^ Computational models likely will yield even further insights as they are applied to DEL selection data.^20^

In this paper, we introduce a method for analyzing DEL selection data at the building block level, with the goal of gaining insights that we can use to design better DELs for subsequent screening rounds. First, we introduce an interpretable analysis of the individual building blocks. Second, we quantify how building blocks interact with each other to determine whether a compound binds to the target of interest. Third, leveraging the idea that similar compounds have similar properties,^21^ we demonstrate how we can use similarity scoring methods to predict the productivity of new building blocks and how similarity metrics differ in their ability to do so. Finally, we build a model that combines the behavior of building blocks at each position into a statistical prediction on the probability of an untested molecule binding to the target of interest.

We note that all of the results in this paper come from a pooled set of three-cycle OpenDEL libraries from HitGen screened against a single-target, soluble epoxide hydrolase (sEH). We release all of the data we analyzed in this study so that interested researchers are able to reproduce our findings. We emphasize that while the findings presented here are specific to this set of DELs on sEH, we believe that our informatics workflow can be extended to analyze the results of various DEL campaigns.

## Results and Discussion

We begin by defining the idea of **productivity** for individual building blocks, which we use to assess whether an overall compound binds to a target. We demonstrate how quantifying the productivity of individual building blocks can provide general insights into structures that could contribute to binding of a target of interest. We then developed a method to guide subsequent DEL screens on a target by (1) identifying productive candidates from a list of proposed building blocks and (2) predicting whether compounds containing those identified building blocks bind to the target of interest. We demonstrate this concept by splitting our data into training and holdout sets (where the holdout sets contain building blocks not seen in training) and provide a workflow for how to incorporate this method in a practical setting.

### Building Block Metric, P(bind), Identifies the Most Productive Building Blocks at Each Position

This section introduces a metric that we call P(bind) to quantify the productivity of building blocks from a set of DEL selection data.

#### Notation for Building Block Positions

**Figure 1:**
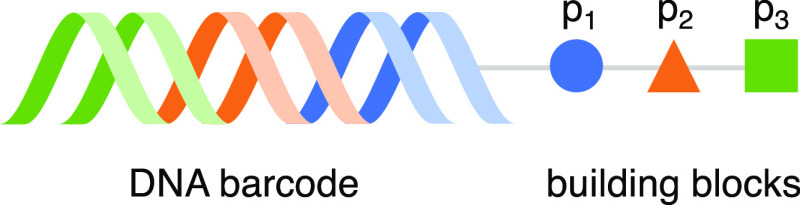
Schematic of the DEL library members. All DEL library members in this study are composed of three small-molecule building blocks and referred to as trisynthons. The first added building block is closest to the DNA (position 1) and the last added is furthest (position 3). Each building block has a corresponding DNA tag encoding its identity, shown in this figure via color coordination. The combined DNA tags form a unique barcode, which is amplified and sequenced in the experiment to verify the presence of the trisynthon. Pictured are position 1 (blue), position 2 (orange), and position 3 (green), which we refer to as *p*_1_, *p*_2_, and *p*_3_, respectively.

To aid in interpretation, we establish a bit of notation. For building block positions, we refer to the position closest to the DNA tag as *p*_1_, the middle position as *p*_2_, and the position furthest from the DNA tag as *p*_3_ (Figure 1). Each of these building block positions is called a **monosynthon**. We denote the set of all building blocks for a given position as *BB*_*i*_, where 1 ≤ *i* ≤ 3 in this study. Individual building blocks are denoted as *bb*_*x*_, where *x* is the identifier, ID, assigned to each unique building block. We refer to two building block positions considered jointly, also known as a **disynthon**, using the notation *BB*_*i*_*BB*_*j*_. In our definition, the two positions considered for a disynthon do not need to be adjacent. Finally, we denote **trisynthons** as *BB*_1_*BB*_2_*BB*_3_. To specify a subset, we use a vertical bar from set building notation^22^ where subset conditions are to the right of the bar. For example, {*BB*_1_*BB*_2_ | *BB*_1_ = *bb*_1_} represents the set of disynthons where position one contains the building block with ID 1.

#### Each Position in the Library Contains a Small Number of Highly Productive Building Blocks

First, to compare building blocks quantitatively, we require a metric to characterize a desirable versus undesirable building block. We define the productivity of a building block, **P(bind)**, as the fraction of compounds that bind to the target when a given building block occurs in a particular position. In this study, we defined binders as compounds with a read count statistically different from 0 at a 95% confidence threshold, making the assumption that read counts follow a Poisson distribution^16^ (see the Methods section for more details).

To illustrate how we calculate P(bind), we provide the following example. Let *S* be the subset of trisynthons such that position *p*_1_ contains the arbitrary building block *bb*_*x*_. This would be expressed as
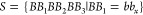
1If the number of trisynthons in the subset *S* is *N*, the P(bind) of the building block *bb*_*x*_ is
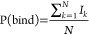
2where *I*_*k*_ is 1 if the *k*th compound in *S* binds to the target and 0 otherwise, as defined in the Data Curation section. We repeated this calculation by changing the subset represented in eq 1 for each building block in each position of the library. Comparing building blocks by their P(bind) values then allows us to identify the most productive building blocks for each position.

**Figure 2:**
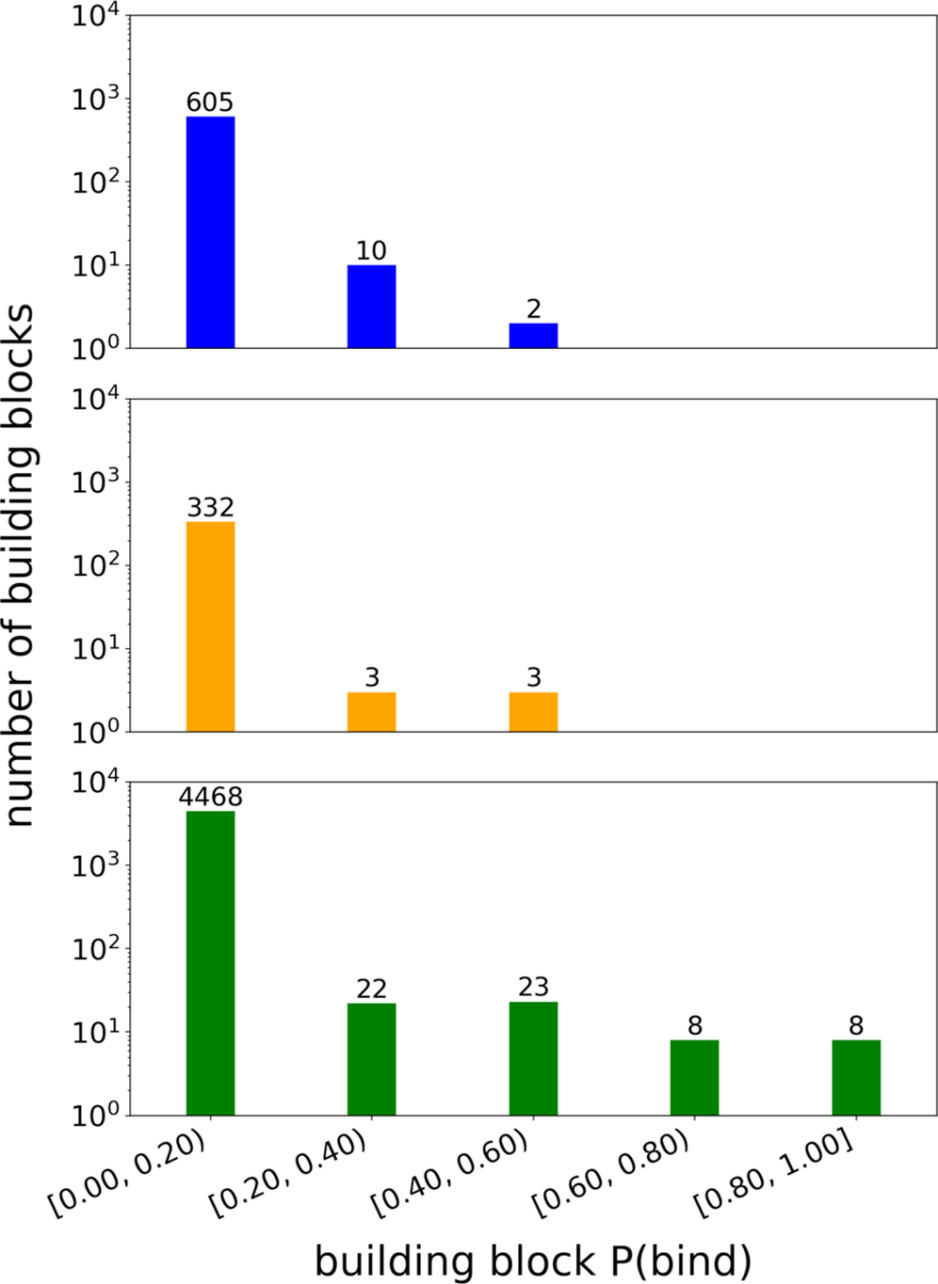
Distributions of P(bind) values for each building block position. Building blocks at each position are separated into P(bind) bins, with the value above each bar indicating the number of building blocks contained in each interval. Shown are the distributions of P(bind) values for building blocks in *p*_1_ (blue, top), *p*_2_ (orange, middle), and *p*_3_ (green, bottom).

We identify a small fraction of building blocks in each position with P(bind) values significantly higher than average. Splitting building blocks into intervals based on their P(bind) values, we find that the distribution of P(bind) at every position is heavily right-skewed, with more than 95% of building blocks at every position having P(bind) values less than 0.20 (Figure 2). For positions 1 and 2, the top 1% of P(bind) values are contained in the P(bind) interval [0.40, 0.60), whereas for position 3, the top 1% of P(bind) values extends across the P(bind) interval [0.80, 1.00]. Given the mean P(bind) value of all building blocks at each position is on the order of 10^–2^, this tells us that the top building blocks occur in binders at a rate about 50 times higher than average.

In this analysis, the difference in the maximum P(bind) value between one position and another reveals that trisynthons are more sensitive to the building block present at certain positions. We posit that in this DEL where trisynthons are synthesized linearly (Figure 1), position 3, being the furthest from the DNA barcode and therefore the most exposed, has the greatest effect on whether the compound binds. Position 1 has the smallest effect on overall compound binding as the position closest to the DNA tag. Since DELs are typically screened with DNA tags still attached, we believe the presence of the DNA tag may partially obstruct interactions with the target. We note that our observation of the importance of position 3 could also be confounded by a larger and more diverse selection of building blocks in that position. However, with the exception of 10 building blocks in position 3, every building block in each of the three positions is used in a statistically significant number of compounds (*N* > 30)^23^ (Figure S1). This suggests that our calculation of the P(bind) metric for each building block should not be highly impacted by small sample sizes. Thus, we believe that when we observe building blocks with high P(bind) values, these values indicate the building blocks are truly productive rather than having values that appear high as an artifact of sampling bias.

It is certainly possible that our finding that building block productivity varies based on library position also points to an issue with false negatives in DELs. Due to the high-throughput nature of DEL screens, it has been demonstrated that larger library sizes lead to high false negative rates.^10^ However, we believe that because the P(bind) metric is aggregated across all of the compounds in which the BB occurs, the metric should be more robust to false negatives. Moreover, we observe some alignment between building blocks we find to be most productive in position 3 and structural motifs of sEH inhibitors in the literature. Notably, the top two most productive building blocks in position 3 resemble benzhydryl pharmacophores that have been reported in the literature to form favorable pi-stacking interactions with residues in the binding pocket of sEH (Figure S2).^24^

When various physicochemical properties of more and less productive building blocks are compared at each position, we find some commonalities. For example, the most productive building blocks in all positions have higher calculated logP. The most productive building blocks in positions 1 and 2 are also characterized by fewer hydrogen bond donors, whereas the most productive building blocks in position 3 have fewer hydrogen bond acceptors and more hydrogen bond donors than their less productive counterparts (Figure S3). We note that our method may be able to broadly detect target-specific architectures that are favored for binding (as in this case with sEH) based on the differences in productivity for the building blocks in different positions.

#### Building Block Productivity Increases the Variety of Binding Disynthon Pairs

Having identified productive building blocks at each position, we proceeded to investigate what characterizes a building block with a high P(bind) value chemically. To do so, we analyze how building blocks combine at a disynthon (pairwise) level. We hypothesize that building blocks with high P(bind) values are **compatible** with a greater number of other building blocks. Here, we define two building blocks as compatible if they co-occur in a compound that binds to sEH.

To test our hypothesis, we evaluate how the number of compatible partners for a building block varies with the P(bind) value of the building block. To calculate the number of compatible partners, we first identify all compounds that bind when a building block is in a certain position. We then count how many unique building blocks are in the other two positions on this list of binders. The number of compatible building blocks in position *p*_*j*_ for an arbitrary building block *x* in position *p*_*i*_ can then be expressed as
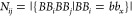
3where the vertical bars on each side of the subset are the cardinality or number of elements in the subset.^22^

**Figure 3:**
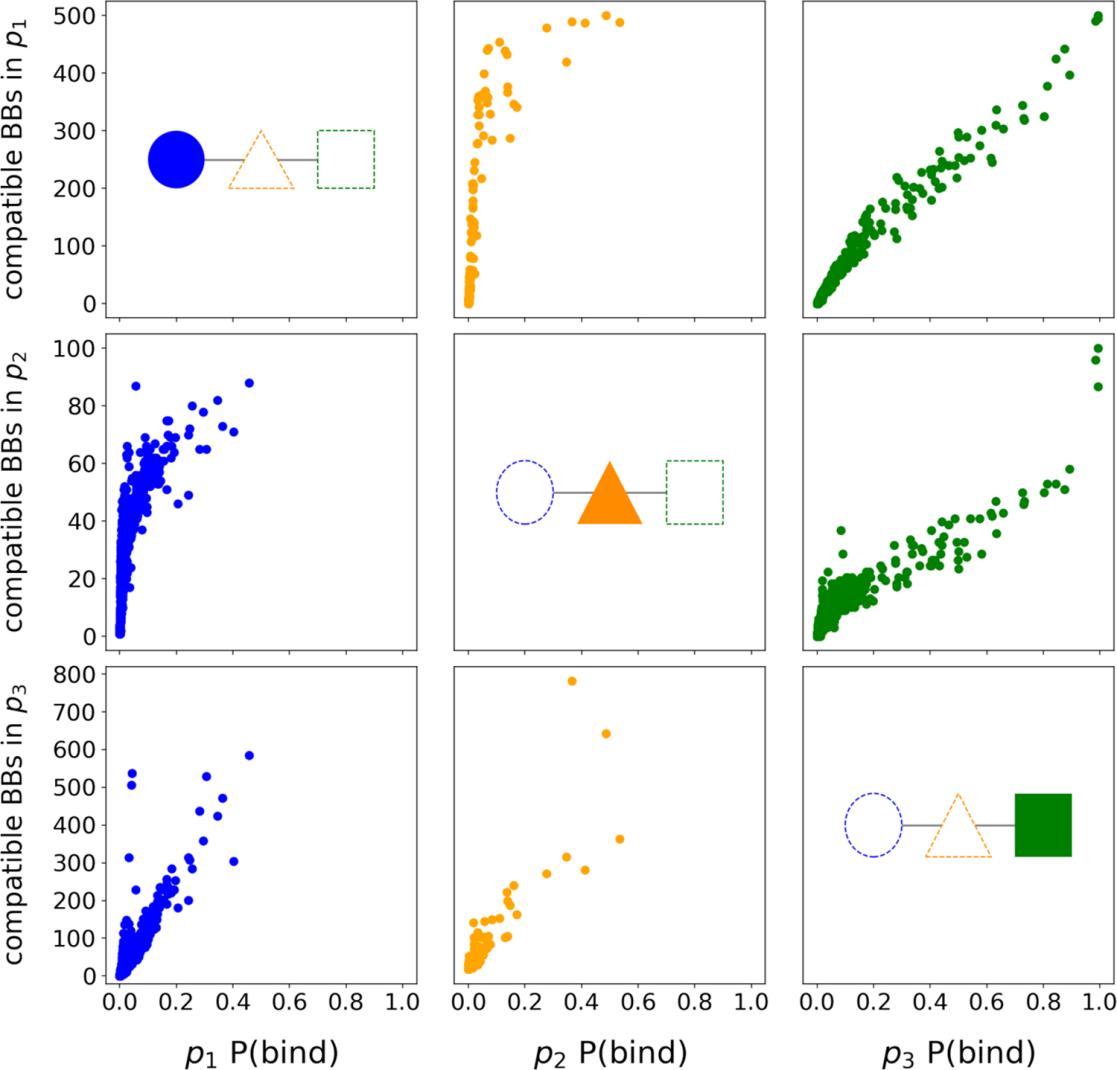
Number of compatible partners as a function of P(bind) for each building block. Building blocks are called compatible if they are present together in a compound that binds. Each column shows how as the P(bind) of the building block in one position changes (filled shape), so does the number of compatible building blocks in the other two positions (dotted shapes). Shown are the results when building blocks in *p*_1_ (left column), *p*_2_ (middle column), and *p*_3_ (right column) are taken as reference.

We find that high P(bind) building blocks form binders with a broader range of partners in both positions. We observe a monotonically increasing relationship between P(bind) and the number of compatible partners for all building block pairs (Figure 3). P(bind) tells us how successful a building block is when it is placed in a certain position, but tells us nothing about the behavior at other positions that may lead to the success (or lack thereof) at one position. Hypothetically, a building block could have a high P(bind) value but only form binders with a very limited selection of partners in one of the other positions. To illustrate this possibility, imagine a scenario where all binders that contain *bb*_*x*_ in *p*_3_ occur only if one or a few specific building blocks are present in *p*_2_. This would attribute all of the variation between these compounds to the identity of the building block in *p*_1_. Since we find the least sensitivity to molecule binding in *p*_1_ (Figure 2), this is a realistic hypothesis to rule out.

**Figure 4:**
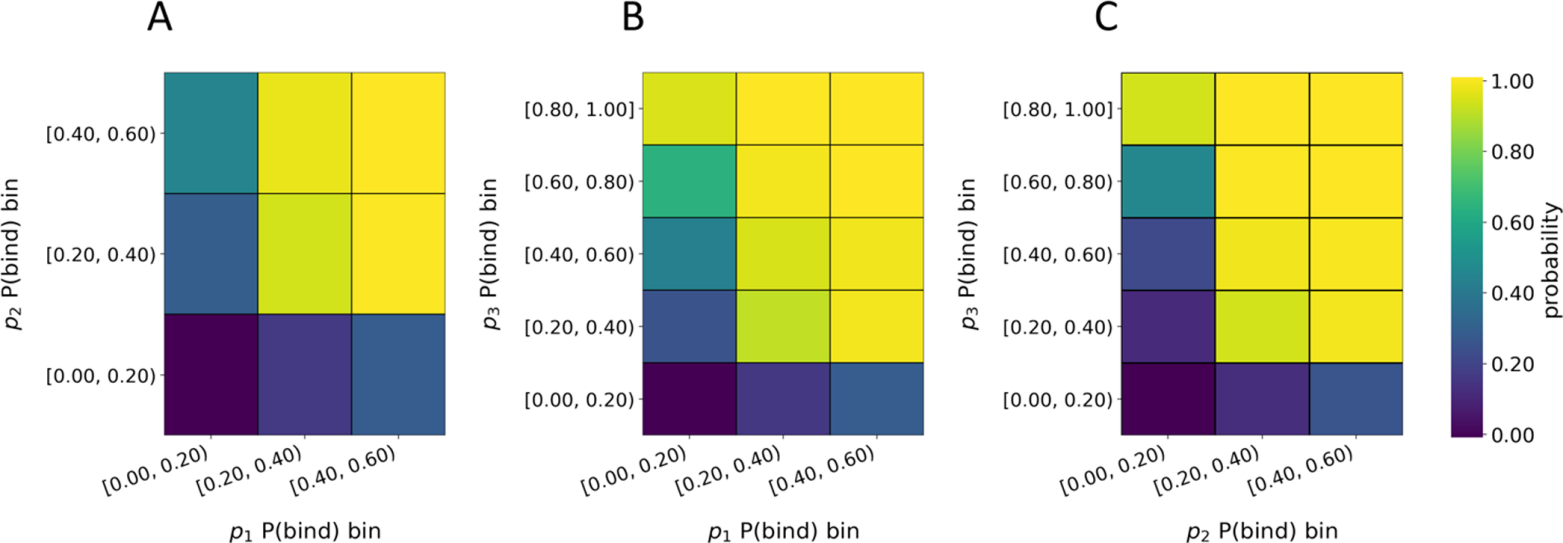
Joint probability of forming a binder using P(bind) bins. The P(bind) bins for each position are the same, but *p*_3_ has more bins because its building blocks span a wider range of P(bind) values. Pictured are the joint probabilities of forming binders from building blocks in bins of (A) *p*_1_ and *p*_2_, (B) *p*_1_ and *p*_3_, and (C) *p*_2_ and *p*_3_.

On the contrary, we see that building blocks that are successful in one position are compatible with a broader diversity of building blocks in all other positions. We note that this could partially be attributed to variations in coupling reactions present in our library, which we address later in our discussion. Previous work has shown that in DELs, reactions are more or less prevalent based on their compatibility with the available building blocks rather than their perceived robustness in traditional settings.^25^ However, what this analysis determines is that we can generally be more confident that a compound containing an untested building block is more likely to bind to sEH if it contains a high P(bind) building block in any position (Figure 4A–C, Tables S1–S3).

### Evaluating the P(bind) of Building Blocks Jointly Predicts the Binding of Trisynthons

In the following section, we transition to analyzing DEL selection data at the trisynthon level. We quantify the probability of forming binders by combining building blocks with varying P(bind) values. We note here that while we only demonstrate this analysis on 3-cycle DEL data in this work, we believe our methodology can be applied to DELs of various cycle numbers in order to identify productive BBs at each position. For 2-cycle DELs, we would only need to consider the interaction between a single pair of positions, which we believe would simplify the analysis.

#### Higher P(bind) in Individual Positions Leads to Higher Probability of Molecule Binding

To understand how varying the P(bind) values of building blocks at each position affects the probability of forming a trisynthon that binds, we calculated joint probabilities for pairs of building block positions using the P(bind) bins shown in Figure 2. We refer to bin positions numerically, where 1 is the lowest bin of P(bind) values, [0.00, 0.20), and 5 is the highest bin of P(bind) values, [0.80, 1.00]. The subset of compounds where the building block in *p*_*i*_ is a member of bin_*x*_ (denoted by the set membership symbol ∈)^22^ and the building block in *p*_*j*_ is a member of bin_*y*_ is
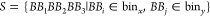
4where 1 ≤ *x*, *y* ≤ 5. We calculate the number of elements in eq 4, *N*, and then use eq 2 to find the joint probability of forming binders for pairs of building block positions (Figure 4).

The joint probabilities reveal that typically for **disynthon combinations**, or a pair of building block positions, increasing the P(bind) of either building block increases the probability of forming a binder (Figure 4A–C, Tables S1–S3). Furthermore, we find that high P(bind) building blocks can be used to **rescue** binding when combined with building blocks with lower P(bind). The higher the P(bind) of a building block in one position, the lower the P(bind) in another needs to be to achieve the same probability of forming a binder. There is a noticeable increase in the probability of forming a binder when the building blocks in both positions have P(bind) values greater than 0.20 (bin 1) (Figure 4A–C, Tables S1–S3).

We find additional evidence that the building block in position 3 has the greatest effect on trisynthon binding. When the building block in position 3 has a P(bind) value in the range [0.80, 1.00] (bin 5), the probability of forming a binder is never less than 93% (Figure 4B,C, Tables S2 and S3). Building blocks in positions 1 and 2 exhibit far less influence and subsequently do not rescue binding to the same extent that building blocks in position 3 can (Figure 4A, Table S1).

Despite variations in the extent to which each building block position contributes, the general trend is clear: introducing a high P(bind) building block in any position increases the probability of forming a compound that binds to sEH. We find that on average, combining monosynthons constructively increases P(bind) (Figure 4). This means that building blocks that are good independently are still good together on average. While this finding is true on the aggregate, we note that we cannot necessarily propose *a specific* combination of building blocks that includes a high P(bind) building block and expect them to form a binder without considering the chemistry used to form the DEL. For example, the DEL might have used different reactions for linking different categories of building blocks so that one part of the DEL might contain productive building blocks that simply cannot be linked to other building blocks that would require linking via a different reaction. Or, certain building blocks might be hindered from linking due to steric constraints or other reasons—in other words, the linkage is not synthetically accessible. Thus, we raise an important caveat: the results presented are conditional on the fact that a product is and can be formed, i.e., that the product is a result of what we call compatible building blocks.

#### Training on Building Block P(bind) Values Yields Precise Predictions for the Binding of Trisynthons

To determine how much signal the P(bind) value alone has in predicting whether a trisynthon binds, we design a simple test. We randomly split our total data set into a training set containing 90% of the data and a test set with the remaining 10%, while ensuring that all building blocks in the training set are sampled in the test set. This means all of the trisynthons in the test set are strictly new combinations of already tested building blocks, allowing us to evaluate whether P(bind) values can be used to predict if a trisynthon binds when the P(bind) values for each building block can be calculated. In later sections, we tackle the issue of predicting whether trisynthons composed of untested building blocks are binders.

**Figure 5:**
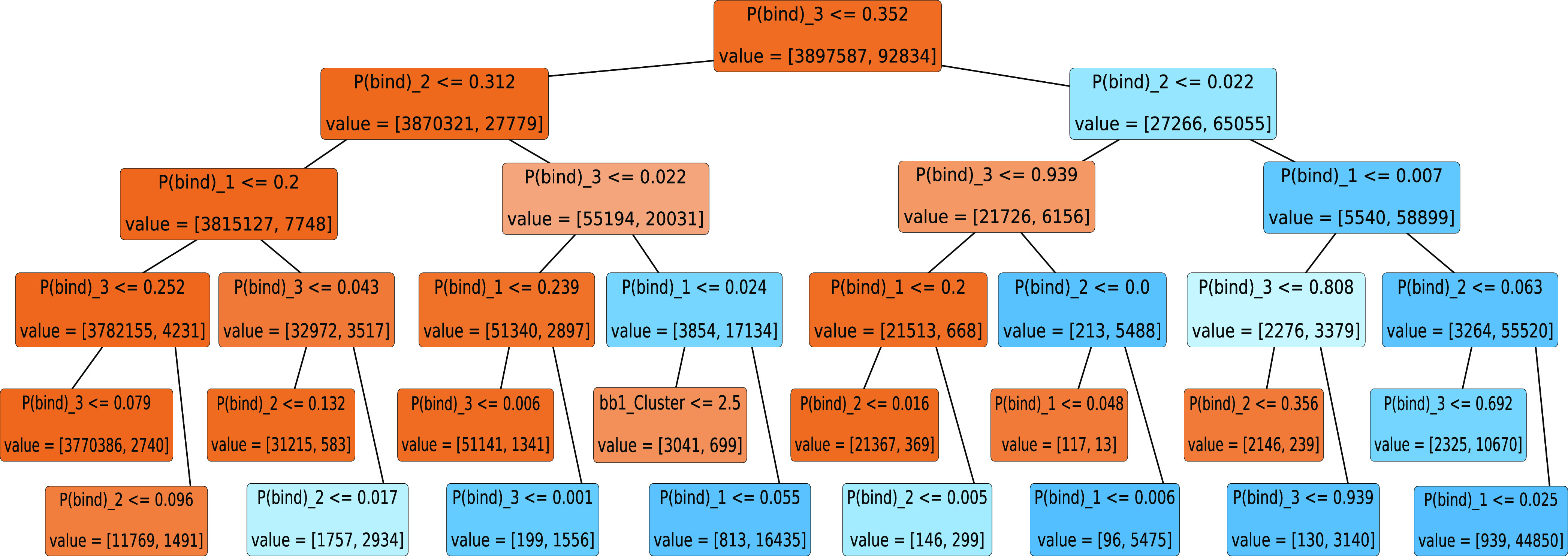
Decision tree based on the P(bind) values at each building block position. Each node, shown as boxes, of the decision tree indicates a split of the data on the condition specified in the first line of text in each box. If the condition is true, the data is split into the bottom left node; otherwise, the data is split to the bottom right node. Darker orange nodes indicate a higher proportion of nonbinders, and darker blue nodes indicate a higher proportion of binders. On the bottom of each node is the value of the number of [nonbinding, binding] compounds.

We find that we can identify trisynthons that bind reliably solely using the P(bind) values of their constituent building blocks. We construct a simple decision tree that splits the data based on the P(bind) value at one of the building block positions (Figure 5) and evaluate the performance of the model using the metrics precision and recall (see the Methods section). Of the 10,302 binders in the test set of 443,380 trisynthons, the decision tree model identifies 9432 true positives and incorrectly predicts 364 false positives, resulting in a test precision of 0.963 () and a test recall of 0.916 (). The area under the curve (AUC) of the precision–recall curve is 0.961, which is significantly higher than the AUC for a random guessing model, which is equal to the hit rate of the test set (). Given that the AUC of a perfect classifier is equal to 1.0, this demonstrates that using building block P(bind) to predict whether a trisynthon binds is highly reliable for this DEL data. A similar analysis can be performed for other DELs and targets to verify the fidelity of this analysis for alternative systems.

We note that our analysis ignores singletons, cases in which species are only enriched in a single selection because we do not analyze the results of multiple DEL selections.^26^ As shown in the literature, some apparent nulls from a single selection have turned out to be high-affinity hits when multiple selections were performed^16^ or when the binding affinity of singletons has been further assessed.^8,27^ Thus, we acknowledge that failing to account for singletons in our analysis could potentially result in an increased false negative rate,^16^ a shortcoming accounted for in other existing methods in the literature.^28^ However, it has also been reported in the literature that singletons are often false positives^26^ and do not exhibit the high affinity that repeat hits do.^29,30^ Historically, DEL practitioners have leaned toward investigating compounds whose neighboring structures show similar behavior,^31^ with the goal of identifying families of related ligands and gaining general insights into structure–activity relationships (SARs) for a target of interest.^8^ We believe that in spite of its inability to address singletons, our method provides a systematic and reproducible way of elucidating general SARs, and offers value as a better alternative than manually evaluating DEL selection data.^31^

### Clustering Based off Chemical Similarity Estimates the P(bind) of Untested Building Blocks

In this section, we discuss how to use similarity scoring to predict the P(bind) value of building blocks that have not been tested, allowing us to extend the applicability of our method to new data.

#### Building Blocks with Similar P(bind) Are Close to Each Other in Projections of Chemical Space

**Figure 6:**
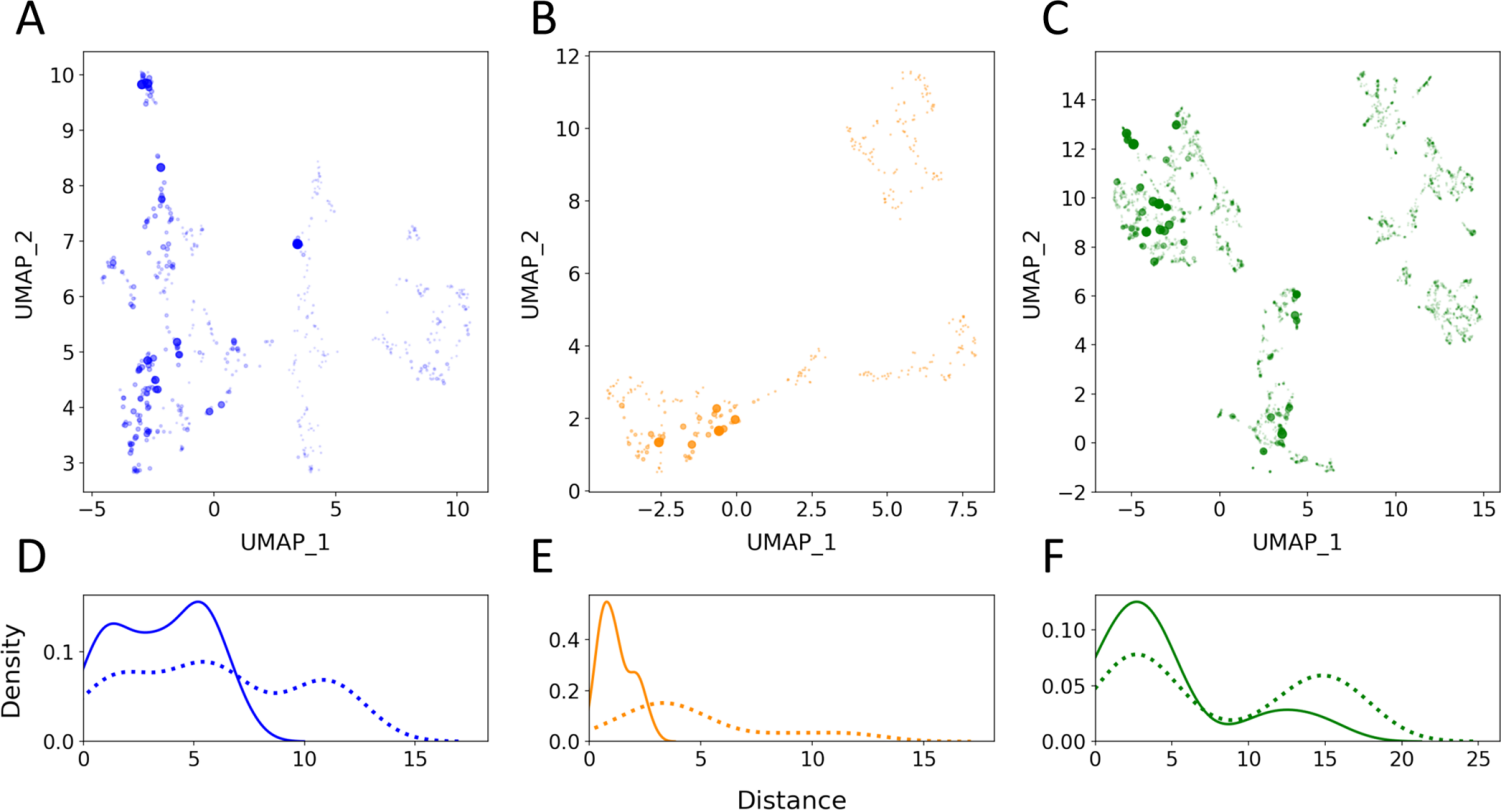
(A–C) UMAP projection of chemical space for each library position. The relative chemical distance between building blocks at each position is represented by the distance between points in the UMAP projections. The size and transparency of each point are scaled by the P(bind) of the building block, with larger, solid color dots indicating building blocks with higher P(bind) values. Pictured are the building blocks in (A) *p*_1_, (B) *p*_2_, and (C) *p*_3_. (D–F) Distributions of distances in UMAP space between the top 10 building blocks by P(bind) and randomly selected building blocks. Pictured are the distances between top 10 to top 10 (solid line) and top 10 to random (dotted line) building blocks for (D) *p*_1_, (E) *p*_2_, and (F) *p*_3_.

We hypothesize that by the **similar property principle**,^21^ building blocks that are similar to each other will have similar P(bind) values.^32,33^ In this study, we elect to use a combination of three-dimensional (3D) shape and color Tanimoto, otherwise known as Tanimoto combo as our similarity metric.^34,35^ For each position, we calculate the Tanimoto combo scores between all building blocks and transform these scores into two-dimensional (2D) coordinates via Uniform Manifold Approximation and Projection (UMAP), a dimensionality reduction technique.^36,37^ Using the UMAP coordinates, we create an approximation of chemical space, where each building block is represented by a point, and the Euclidean distance between points is inversely proportional to the chemical similarity of the respective building blocks (Figure 6A–C). We emphasize that no information regarding the P(bind) value of building blocks is introduced in this process.

We find that high P(bind) building blocks generally are much closer (and therefore more similar) to one another than they are to random building blocks (Figure 6D–F and Table S4). Here, we define high P(bind) building blocks as the top 10 by descending P(bind) value at each position. On average, the Euclidean distance from a high P(bind) to a randomly selected building block is twice as large as the distance from one high P(bind) building block to another (Figure 6D–F and Table S4). This supports our hypothesis that similar building blocks have similar P(bind) values and motivates our next step: to predict the P(bind) value of an untested building block based on the P(bind) values of the building block(s) most similar to it.

We also test if 2D or 3D Tanimoto similarity results in a clearer separation of clusters. We observe more random separation between building blocks of similar P(bind) value when using 2D Tanimoto similarity instead of 3D Tanimoto combo (Figure S4). The UMAP projections from 2D Tanimoto show building blocks with similar P(bind) value scattered throughout chemical space with less structure (Figure S4, Table S5). A potential explanation for this is because 3D Tanimoto takes into account multiconformer overlays of 3D structures, it is able to better relate the binding ability of molecules compared to 2D Tanimoto.

#### Groupings by Chemical Similarity Are Predictive of Building Block Binding

**Figure 7:**
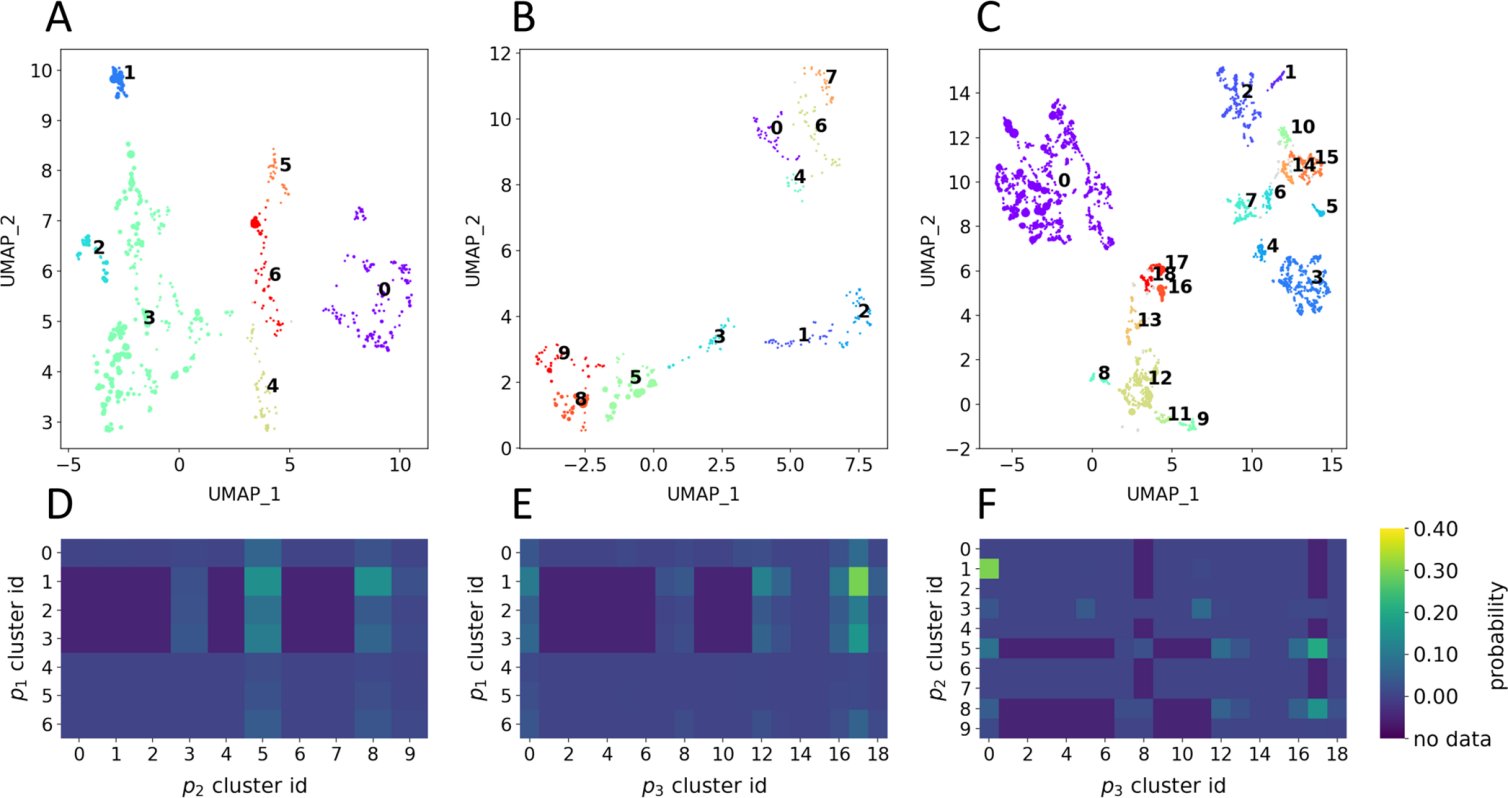
HDBSCAN clusters on UMAP projection of each library position. (A–C) We apply HDBSCAN to the UMAP projections of each library position in order to group similar building blocks into clusters. Each cluster is identified visually by a different color and assigned a numeric cluster ID. Pictured are the cluster assignments for (A) *p*_1_, (B) *p*_2_, and (C) *p*_3_. (D–F) Joint probability of forming binders using HDBSCAN clusters. Aggregating trisynthon data by cluster ID allows us to identify which combinations of building blocks have a high and low probability of forming binders. We also indicate combinations of building blocks that are never observed in the data. Shown are joint probabilities when combining clusters from (D) *p*_1_ and *p*_2_, (E) *p*_1_ and *p*_3_, and (F) *p*_2_ and *p*_3_.

**Figure 8:**
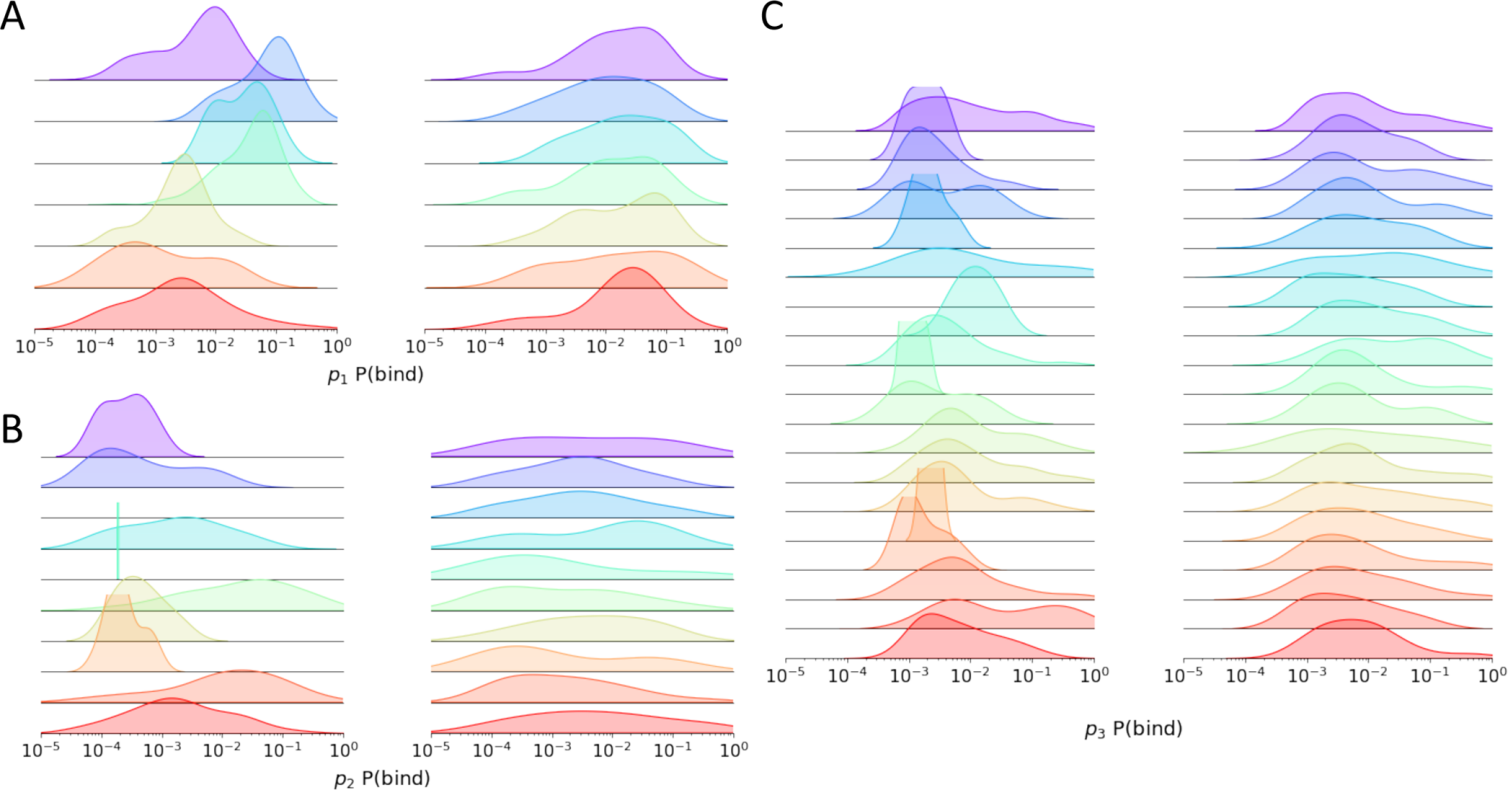
Distribution of the P(bind) values for clusters. We visualize the distribution of P(bind) values for clusters formed via HDBSCAN (left) and clusters formed from randomly selecting compounds (right). The color of each cluster matches the color assignments in Figure 7. To better visualize each distribution, we remove all building blocks where P(bind) = 0 and plot P(bind) values on a log scale. Empty grids indicate clusters where all members have P(bind) = 0. Shown are the results for (A) *p*_1_, (B) *p*_2_, and (C) *p*_3_.

We find that we can form clusters to estimate the P(bind) value of untested building blocks. To do so, we first apply HDBSCAN^38,39^ to the UMAP coordinates of building blocks (Figure 6A–C), resulting in a set of clusters for each position in projected chemical space (Figure 7A–C). After assigning clusters, we compare the full width at half-maximum (fwhm)^40^ of the distribution of P(bind) values for HDBSCAN-generated clusters to randomly generated clusters. We find that the average fwhm of the P(bind) distributions from HDBSCAN-generated clusters is less than that for random clustering (Figure S5), showing that compounds tend to be grouped into clusters of somewhat similar P(bind) values. Thus, we conclude that using a building block’s cluster assignment to predict its P(bind) value (Figure 8) improves accuracy compared to random guessing.

Beyond predicting the P(bind) of untested building blocks, clusters can also be used to identify groups of building blocks that are compatible. After assigning each building block to a cluster, we join the cluster results on the trisynthon data in order to get a list of three cluster IDs (corresponding to the cluster assignment for the building block at each position) for each trisynthon. Grouping by the cluster ID at each position then allows us to calculate the probability of forming compounds that bind to the target for every distinct cluster combination (Figure 7D–F, Tables S6–S8). We can describe the subset of compounds where the building blocks in positions *p*_*i*_ and *p*_*j*_ are members of the *x*th cluster of *p*_*i*_ and the *y*th cluster of *p*_*j*_ as
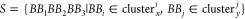
5As before, we calculate the joint probability for disynthons by calculating the number of entries in *S*, *N*, and then we apply eq 2.

Moreover, we also identify certain combinations of clusters that are not observed in the experimental data (Figure 7D–F, Tables S6–S8). While the analysis does not indicate why these combinations of building blocks are not observed, we believe that characterizing these gaps could be useful. For example, gaps could be new combinations of building blocks that might be desirable to test. On the other hand, these gaps could also indicate that the combination can not be made (e.g., due to a DEL being formed using several different reactions so that certain building blocks cannot be cross-linked given the reactions employed) or that something went wrong experimentally so that even though the combination was thought to be tested, no data was collected. Thus, in some cases, gaps in the data may represent building blocks or combinations of building blocks to avoid and, in others, areas to test in further rounds of experimentation.

### Application to Holdout Data

In this final section, we demonstrate how we would apply this method in a practical setting, where we would work to guide the design of a new DEL using information available from a prior screen. Here, we model this design process by testing the performance of our model using a holdout set (using building blocks not seen previously) to mimic a new set of building blocks to test.

#### Building Block Level Analysis Predicts Whether Trisynthons Containing Untested Building Blocks Bind to sEH

To simulate an experimental setting in which we would like to choose promising new building blocks to study after performing an initial set of DEL screen(s), we randomly select 5% of the building blocks at each position, remove all trisynthons containing any of those building blocks, and place them into a holdout set. The training set, now composed of the remaining compounds, represents the information we might have obtained after an initial experimental screen that had used only a limited set of building blocks. The holdout set represents a set of proposed follow-up candidates that contain at least one untested building block.

**Figure 9:**
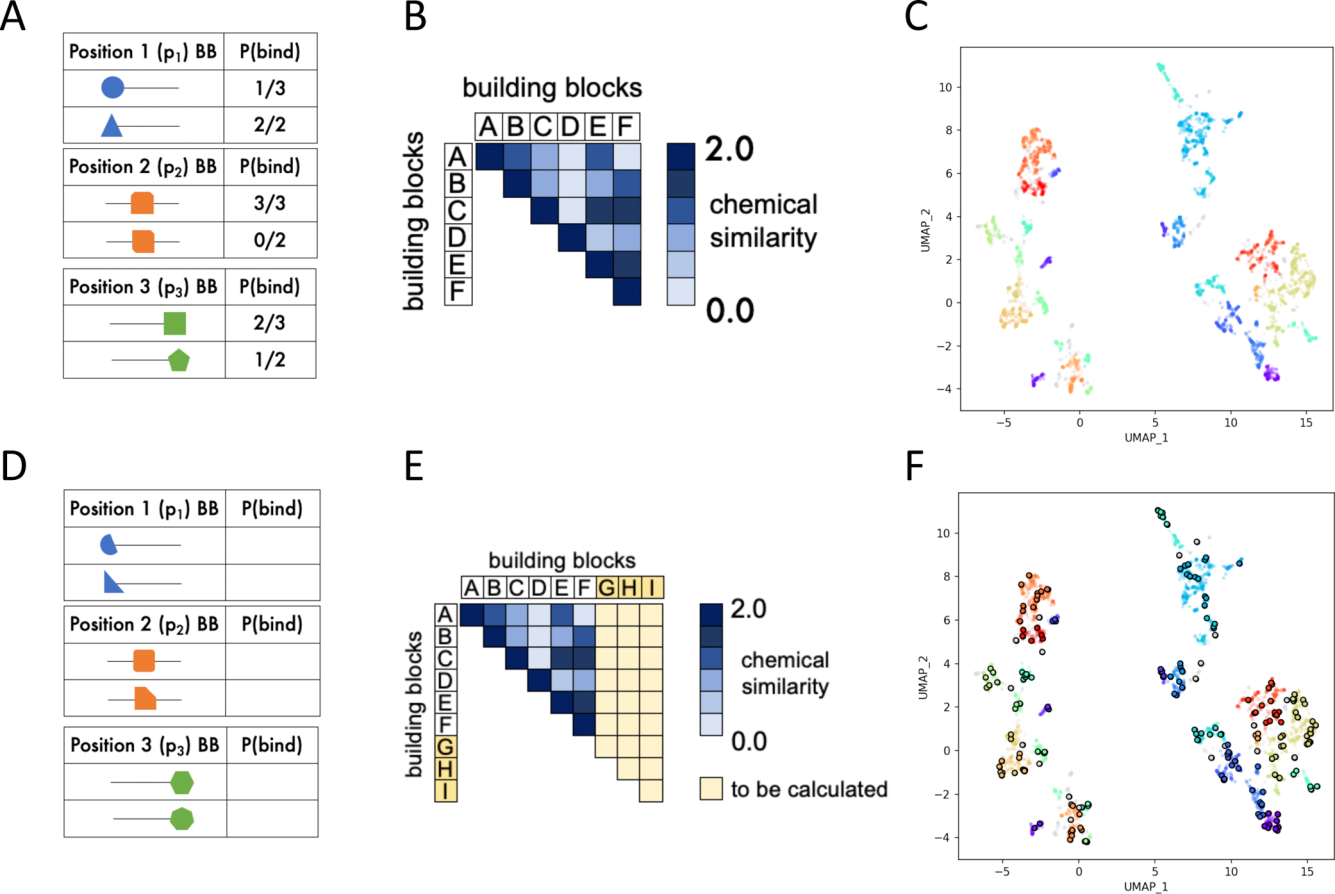
Overview of protocol to predict the productivity of out-of-sample building blocks. (A–C) Protocol for processing existing DEL selection data. (A) We calculate the P(bind) metric for all of the building blocks in the library. (B) We compute the 3D Tanimoto combo between all of the building blocks at each position in the library. (C) We transform similarity scores among building blocks into a mapping of chemical space via UMAP and resolve clusters with HDBSCAN. (D–F) Protocol to apply our methodology to new proposed building blocks. (D) We propose a set of building blocks that have not been tested experimentally. (E) For each position separately, we calculate the 3D Tanimoto combo between the new set of building blocks and the existing ones. (F) We map the new building blocks onto the existing UMAP projections and assign each one to a cluster.

Our workflow proceeds as follows: (1) Calculate the P(bind) values for all of the building blocks in our training set (Figure 9A). This P(bind) information is used to train a decision tree classifier to predict whether compounds bind to the target of interest. We can also visualize the most productive building blocks at each position to get a sense of what sorts of chemistries may be favored for binding to the target of interest. (2) Compute the 3D Tanimoto combo among all of the building blocks at each position (Figure 9B). (3) Apply UMAP to each similarity matrix to create a mapping of chemical space for the building blocks at each position and cluster with HDBSCAN to resolve groups of similar building blocks with similar P(bind) values (Figure 9C). Peeking into the building blocks in each cluster can further elucidate structures that are potentially favorable for binding to the target, and aggregating by cluster ID can identify combinations of building blocks at each position that are more and less likely to result in a binder. (4) Identify a new set of building blocks to mix in combination with already tested ones (Figure 9D). (5) Calculate the 3D Tanimoto combo between all of the training set building blocks and a new set of building blocks (Figure 9E). (6) Map new building blocks onto the existing UMAP embedding and classify them into the existing clusters (Figure 9F). (7) Predict the P(bind) of each building block in the holdout set using the building blocks in its cluster. We explore four different methods of approximating the P(bind) value of each building block in the holdout set:

- the median P(bind) of the cluster
- the mean P(bind) of the cluster
- P(bind) of randomly selected BBs from the cluster
- P(bind) of most similar BBs in the cluster

**Figure 10:**
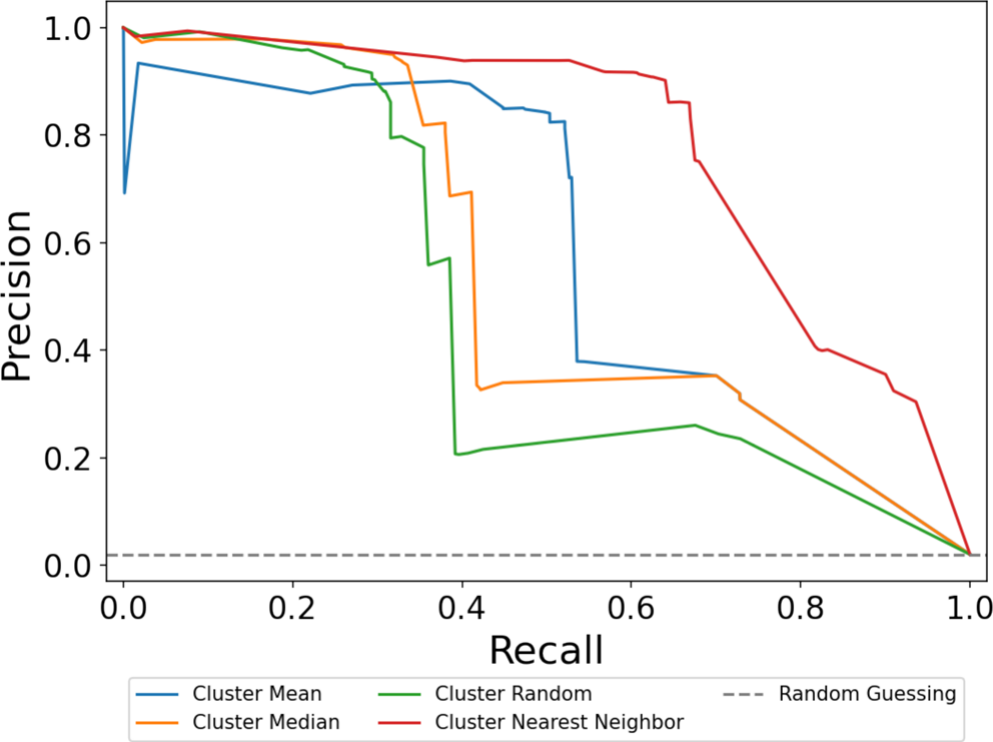
Comparison of the AUC of the precision–recall curves for different prediction methods. We evaluate four different ways to estimate the P(bind) of untested building blocks from HDBSCAN clusters. Predictions are made on a test set, where each compound contains at least one building block not seen in the training set. The random guessing benchmark is equal to the hit rate of the holdout set, which is approximately 2%.

We train a decision tree classifier using the P(bind) and cluster information on every building block in the training set as input (training precision: 0.960, training recall: 0.926) and then apply the classifier to our holdout set. We find that every method outperforms random guessing by at least an order of magnitude (Figure 10). In addition, using the cluster nearest neighbor to approximate untested building block P(bind) gives the best result for predicting the binding of trisynthons to sEH (AUC: 0.799; averaged over 50 random trials; Figure S6). This finding further supports the argument that on average, chemically similar compounds have a greater probability of similar productivity.^32,33^

### Building Block Analysis Identifies Productive Regions of Chemical Space to Probe for Subsequent Screening Rounds

Compared to existing methods in the literature such as the tagFinder^28^ and deldenoiser,^13^ one distinct advantage of our method is the ability to take pooled DEL data screened against a particular target of interest and identify new combinations of building blocks that are more or less likely to form binders for this target. We imagine this can greatly inform subsequent screening rounds, empowering researchers to either exploit combinations of building blocks that are conducive to forming binders or explore different regions of chemical space to build up a diverse assortment of compounds that bind a target of interest. While we only predict the behavior of building blocks that are similar to existing ones, we imagine that because we combine these building blocks in new combinations we can build up a diverse set of final products that are likely to bind to the target. As an illustrative example, we showcase a diverse set of compounds that our method successfully determined bind to sEH (Figure S7).

Using similarity scoring and a decision tree model, we predict binders from a set of compounds containing building blocks not seen in the training set at a rate of more than an order of magnitude greater than random. The performance of this approach demonstrates that even relatively simple models can estimate whether new trisynthons containing building blocks similar to previously tested ones will bind to sEH. As the intersection between machine learning and DELs grows, we challenge researchers to pay attention to straightforward models such as the one employed here and evaluate whether more complex machine learning methods perform significantly better.

## Conclusions

In this work, we applied computational modeling to understand the productivity of building blocks in a set of DELs and predicted how individual building blocks can be combined to form compounds that are likely to bind to a single-target, soluble epoxide hydrolase (sEH). We developed a simple and interpretable method to predict the behavior of new building blocks, their interactions with known building blocks, and whether compounds consisting of holdout set building blocks would bind to sEH.

Our model can be an effective baseline for future studies due to its high accuracy and relative simplicity. In the future, it may be interesting to explore the relative merits of more complex deep learning architectures versus similarity-based methods. For example, approaches employed here may have similar performance to more complex neural networks if out-of-sample data resemble existing data, such as during medicinal chemistry efforts or when only small numbers of building blocks and compounds have been explored. On the other hand, when the amount of available data becomes large, it seems likely that deep learning models perform better.

Given the promise of DEL screens for high-throughput testing of ideas for drug discovery,^13,15,18,19^ the refinement of subsequent DEL screens to minimize cost and enhance follow-up on promising structures is likely to improve outcomes in drug discovery. While the ability to gather a vast variety of data in a DEL screen is an experimental advantage, the volume of data poses challenges for interpretation. Improved computational methods are pertinent to aid the experimental workflow. Our method and open-source software^41^ can be applied to experimental DEL screens in the future to guide building block selection, identify essential features needed to bind the target of interest, and reduce the search space when following up on potential binders.

## Methods

### Data Collection

The data set was generated from in-house screening of several commercially available DEL libraries (OpenDEL from HitGen) against soluble epoxide hydrolase (sEH). The DEL screen was performed as previously described in Clark et al.^8^ Briefly, N-term his-tagged human Soluble Epoxide Hydrolase (sEH) protein (1 μM, N-term His-tagged) was incubated with pooled DEL libraries in a 100 uL reaction (50 mM HEPES (pH 7.4); 150 mM NaCl; 0.01% Tween-20; 10 mM Imidazole; 1 mM TCEP; 0.1 mg/mL ssDNA). Postincubation, the protein was captured by magnetic beads (Invitrogen Dynabeads His-Tag, and Pierce Ni-NTA magnetic beads), and the samples were washed with buffer. Each round of selection was completed by a heat elution (95 °C for 10 min) to separate protein from bound molecules. A new round was initiated by the introduction of fresh protein, and the process was repeated for a total of 3 rounds. In parallel, a matrix-binding-only sample was included to account for nonspecific binding. Postselection, samples were PCR amplified and sequenced on a next-generation sequencing platform.

### Data Curation

We compiled input files after experiment as comma-separated values (CSVs) containing the SMILES of each composite structure, its experimentally determined read count, and the SMILES of its constituent building blocks. In some cases, the SMILES strings for building blocks included the protecting groups used during synthesis, which would be removed in the process of constructing full compounds.

As a first step in curation, we classified compounds into binary categories of either “non-binder” or “binder”—with respective labels of 0 and 1—based on whether their NGS sequencing counts (which we call read counts) were statistically different from 0 at a 95% confidence threshold. We assumed read counts were drawn from a Poisson distribution, a treatment used across several studies in the literature.^13,16,18^ Using this definition, we selected the top 10K binders by read count value and a random selection of 10M nonbinders from the total collection of DEL data screened against sEH. The end result of this is that the lowest read count for any compounds classified as a “binder” was 81, which means we only analyze very clear binders from this data set (Figure S8). We note that sEH is a particularly rich target for DELs^4^ and there may not always be such a clear delineation between binders and nonbinders for other targets. In view of this, we include data reporting how the distribution of compounds classified as either a “non-binder” or “binder” to the target changes as we vary the minimum read count threshold (Table S9). We emphasize that our analysis is not on a complete set of DEL selection data but rather a subset of a larger data set.

We further curated input files using pandas (v.1.2.1)^42,43^ to remove duplicate compounds and lines containing fields with null entries. In cases where we had building blocks reported in duplicate with both unspecified and specified stereochemistry, we elected to remove all compounds containing the building block with unspecified stereochemistry. This was the case for fewer than 5% of the building blocks in any of the positions in the library. We also removed compounds with building blocks containing boron, because we could not generate conformers for them due to force field limitations; this library initially had many boron-containing compounds. Furthermore, some building blocks were reported with protecting groups still present. We used ChemDraw (v.17.0) to generate SMIRKS reactions and used the OEChem toolkit (v.2021.1.1)^44^ to deprotect Fmoc, nBoc, methyl ester, and ethyl ester groups on those relevant building blocks. We did this to ensure that the presence of protecting groups would not bias our similarity calculations and because the protecting groups were not present in the final products. After applying the deprotecting functions, we saved all of the unique building blocks at each library position to separate files. Associated code for these steps can be found at https://github.com/MobleyLab/DEL_analysis.

### 2D Tanimoto

We calculated 2D Tanimoto scores using RDKit (v.2020.09.1.0)^45^ by first converting compounds into Morgan fingerprints^46^ with the radius parameter set to 3 bonds.

### 3D Tanimoto Combo

We calculated 3D Tanimoto combo using the FastROCS toolkit (v.2021.1.1)^44^ from OpenEye. The 3D Tanimoto combo score takes into account both volume (shape) and pharmacophore (color) overlap between two molecules to produce an aggregated similarity score. Both the shape and color scores range from 0 to 1, so the 3D Tanimoto combo has a maximum value of 2.

We first generated up to 200 conformers for each of the building blocks in the library using the Omega toolkit (v.2021.1.1).^44^ We maintained the same settings as the defaults in the Classic OMEGA floe on Orion (Spring 2020), but restricted the stereochemistry of input molecules. For building blocks with unspecified stereochemistry, we used the OEFlipper function in Omega to enumerate all possible stereoisomers and generated up to 200 conformers for each of them.

Next, we used FastROCS to generate an all-by-all matrix of 3D Tanimoto combo scores for all of the building blocks (including enumerated stereoisomers) in each library position. We iterated over each conformer of each building block to identify the highest possible shape and color overlap between the pairs of compounds. Thus, each entry (*i*, *j*) of the 3D Tanimoto combo matrix represented the largest possible overlap in both shape and color between any conformer of compound *i* and any conformer of compound *j*. For compounds with multiple stereoisomers, we identified a single stereoisomer that gave the highest similarity to other compounds. To do so, we first enumerated all stereoisomers for the compound and evaluated the similarity of a given stereoisomer to those of all other compounds. We then selected the stereoisomer that gave the highest average similarity to all other compounds and discarded the rest. Associated code for these steps can be found at https://github.com/MobleyLab/DEL_analysis

#### Computational Considerations

It was infeasible to calculate all-by-all similarity matrices for libraries on the order of 10^6^ molecules, as the task would require performing 10^12^ similarity scoring operations. We instead calculated all-by-all similarity matrices for building blocks at each position individually and then evaluated combinatorial effects at a later step in the analysis. This was a much more computationally tractable approach. Additionally, it mimics considerations involved in library design, one of our key interests, where one might want to use knowledge about current building blocks to help design libraries for screening.

### Generating Clusters

We formed clusters based on the 3D Tanimoto combo score of building blocks at each position. First, we transformed 3D Tanimoto combo scores into distances by subtracting each similarity score from 2, the maximum value for the Tanimoto combo. Due to slight variations in the conformer overlay process, distance matrices were not perfectly symmetric, and some diagonal elements (a compound to itself) had distances slightly greater than zero. To symmetrize the distance matrix, we averaged it with its transpose and set the diagonal elements to zero.

We then used Uniform Manifold Approximation and Projection (UMAP) (v.0.5.3)^36,37^ to perform a dimensionality reduction of our data set from 3D to 2D space, both to help with visualization and because we wanted to pick a coordinate space to use for subsequent prediction of properties for new building blocks. This resulted in the conversion of the set of 3D distance matrices into 2D coordinates for each building block. We inputted these coordinates into HDBSCAN (v.0.8.28)^38,39^ to generate clusters for each library position.

We designed and minimized an objective function to determine the optimal number of clusters. Specifically, we arrived at an objective function, *L*
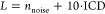
6where *n*_noise_ is the number of points classified as noise and ICD is the average intracluster distance between clustered points for each HDBSCAN run. We performed a grid search over HDBSCAN hyperparameters and calculated the value of the objective function for each set of clusters. We selected the hyperparameters corresponding to the global minimum of the objective function to use for clustering (Figure S10). More information on the design of the objective function can be found in the Supporting Information.

Following the cluster assignment, we predicted the cluster assignment for new building blocks by projecting points onto existing UMAP embeddings and applying the function hdbscan.prediction.approximate_predict. We elected to use UMAP because it was reported to have better performance and reproducibility than other commonly used methods^47^ and was demonstrated to improve the results from clustering algorithms.^48^ Associated code for these steps can be found at https://github.com/MobleyLab/DEL_analysis

### Model Construction and Evaluation

We used Scikit-Learn (v.0.23.2)^49^ to build a decision tree model and assess the quality of model predictions. To reduce the chance of overfitting the training data, we performed 5-fold cross-validation to determine the maximum depth of the decision tree (Figure S11). For our evaluation criteria, we elected to use precision and recall because of the imbalance of class labels in our data set. Recall evaluates the fraction of all true binders that are correctly identified by a classifier and precision evaluates the fraction of true binders from all compounds classified as binders.^50^ For a given imperfect classifier, tuning to yield an increase in precision (better prediction of binders) results in a decrease in recall (fewer binders identified), and vice versa. The quality of a classifier can be described by the extent of this trade-off, which is quantified by the area under the curve (AUC) of the precision–recall curve (PRC).^50,51^ Associated code for these steps can be found at https://github.com/MobleyLab/DEL_analysis.

## Data Availability

All code, data, and results from this study can be found on GitHub at https://github.com/MobleyLab/DEL_analysis and on Zenodo.^41^

## Supporting Information Available

Method to construct the HDBSCAN loss function, data on hyperparameter optimization, results using 2D Tanimoto similarity, and data tables for the figures presented in the main text (PDF)
