# Supplementary material for: Building Block-Based Binding Predictions for DNA-Encoded Libraries
Source: J Chem Inf Model. 2023 Aug 14;63(16):5120–32. doi: 10.1021/acs.jcim.3c00588 (PMC10466377; doi:10.1021/acs.jcim.3c00588)
Supplement: Supplementary file 1 — ci3c00588_si_001.pdf [file ci3c00588_si_001.pdf]

Supporting Information: Building Block-Based Binding Predictions for DNA-Encoded Libraries

Chris Zhang,[†] Mary Pitman,[‡] Anjali Dixit,[‡] Sumudu Leelananda,[¶] Henri Palacci,[¶] Meghan Lawler,[¶] Svetlana Belyanskaya,[¶] LaShadric Grady,[¶] Joe Franklin,[¶] Nicolas Tilmans,[¶] and David L. Mobley[*,†,‡]

†*Department of Chemistry, University of California, Irvine, 1120 Natural Sciences II, Irvine, California 92697, United States*

‡*Department of Pharmaceutical Sciences, University of California, Irvine, 856 Health Sciences Road, Irvine, California 92697, United States*

¶*Anagenex, 20 Maguire Rd Suite 302, Lexington, MA 02421, United States*

E-mail: dmobley@uci.edu

S1 Additional Results

S1.1 Effect of Sampling on Calculation of P(bind) metric

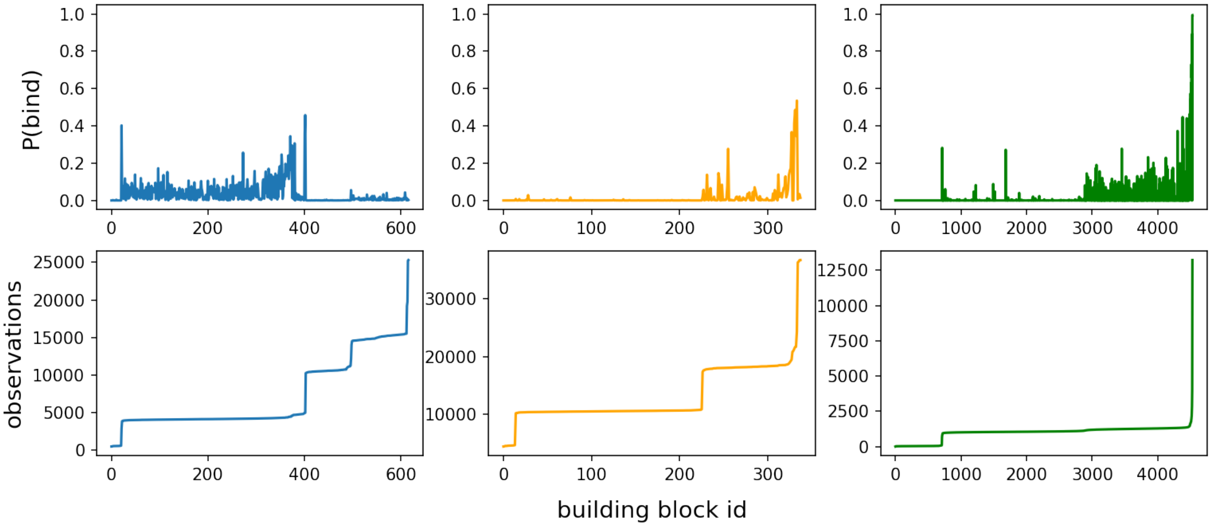

Figure S1: P(bind) value and number of observations for each building block in the library. With the exception of 10 building blocks in position 3, every building block occurs in a statistically significant[1] number of compounds (N>30).

S1.2 Structural Similarities between Productive Fragments and Known sEH Inhibitors

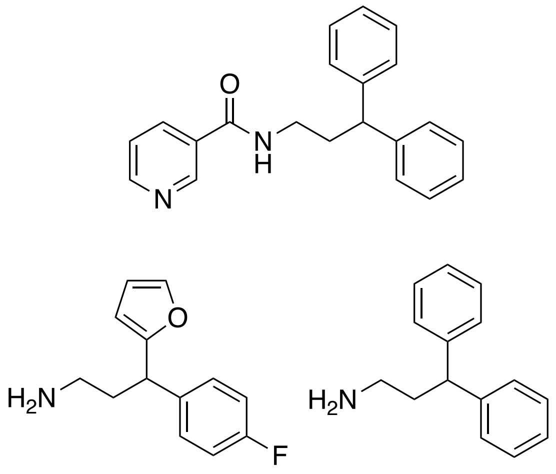

Figure S2: Structure of a known potent inhibitor of sEH from experiment[2] (top) and the structures of the top two most productive building blocks in position 3 identified through P(bind) analysis (bottom).

S1.3 Physicochemical Properties of Productive Building Blocks

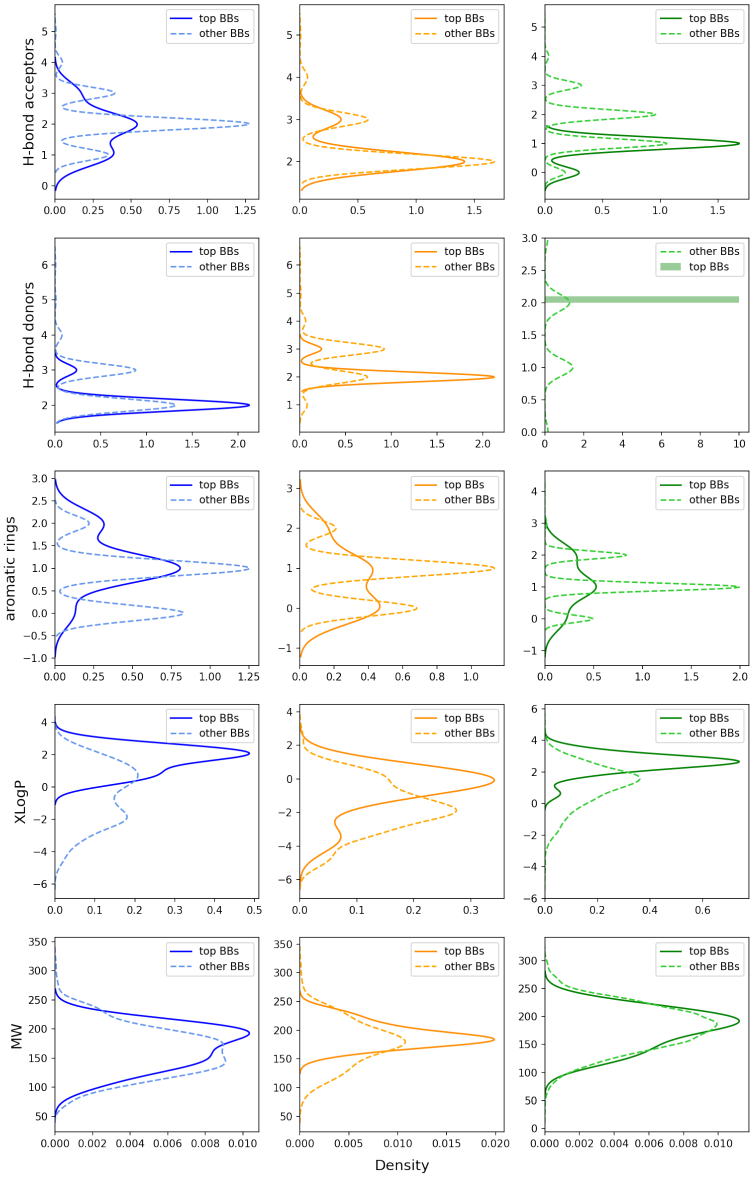

Figure S3: Distribution of physicochemical properties for top building blocks by P(bind) value to all other building blocks at each position. We defined the top building blocks at each position as the top 20 by P(bind) value.

S1.4 Tables of values for pairwise analysis of building block bins

Table S1: Matrix entries for pairwise analysis of building block bins in positions p_1 and p_2

p_2 P(bind) bin	p_1 P(bind) bin		
	$[0.00, 0.20)$	$[0.20, 0.40)$	$[0.40, 0.60)$
$[0.40, 0.60)$	0.4452	0.9451	0.9771
$[0.20, 0.40)$	0.2946	0.9104	0.9697
$[0.00, 0.20)$	0.0100	0.1706	0.2930

Table S2: Matrix entries for pairwise analysis of building block bins in positions p_1 and p_3

p_3 P(bind) bin	p_1 P(bind) bin		
	$[0.00, 0.20)$	$[0.20, 0.40)$	$[0.40, 0.60)$
$[0.80, 1.00]$	0.9417	0.9973	1.0000
$[0.60, 0.80)$	0.6412	0.9813	1.0000
$[0.40, 0.60)$	0.4329	0.9403	0.9768
$[0.20, 0.40)$	0.2579	0.9037	0.9815
$[0.00, 0.20)$	0.0036	0.1666	0.2922

Table S3: Matrix entries for pairwise analysis of building block bins in positions p_2 and p_3

p_3 P(bind) bin	p_2 P(bind) bin		
	$[0.00, 0.20)$	$[0.20, 0.40)$	$[0.40, 0.60)$
$[0.80, 1.00]$	0.9301	0.9994	0.9989
$[0.60, 0.80)$	0.4631	0.9953	0.9981
$[0.40, 0.60)$	0.2261	0.9769	0.9908
$[0.20, 0.40)$	0.1185	0.9301	0.9827
$[0.00, 0.20)$	0.0012	0.1325	0.2630

S1.5 UMAP Projection using 2D Tanimoto Similarity

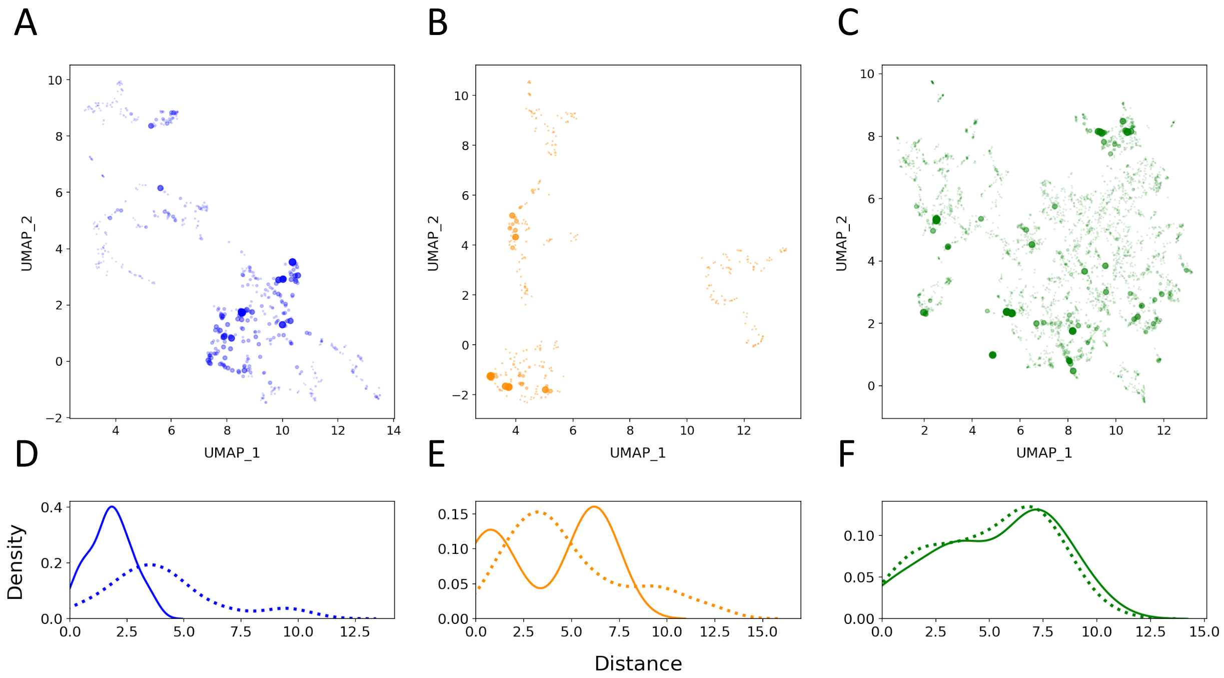

Figure S4: (A–C) UMAP projections of chemical space for each library position using 2D Tanimoto. Pictured are building blocks in (A) p_1, (B) p_2 and (C) p_3. (D–F) Distributions of distances in UMAP space between the top 10 building blocks by P(bind) and randomly selected building blocks. Pictured are the distances between top 10 to top 10 (solid line) and top 10 to random (dotted line) building blocks for (D) p_1, (E) p_2 and (F) p_3. Some separation between high and low P(bind) building blocks still occurs for 2D Tanimoto, but is decreased compared to when using 3D Tanimoto combo.

S1.6 Distances Between Building Blocks in UMAP space

Table S4: Distance between top P(bind) building blocks to other top P(bind) building blocks and to random building blocks using 3D Tanimoto

Position	top - top distance	top - random distance
1	3.4877	6.0072
2	1.2091	4.7872
3	4.6037	8.2252

Table S5: Distance between top P(bind) building blocks to other top P(bind) building blocks and to random building blocks using 2D Tanimoto

Position	top - top distance	top - random distance
1	1.7771	4.0865
2	3.7956	4.8249
3	5.3657	5.1005

S1.7 Table of values for pairwise analysis of building block clusters

Table S6: Matrix entries for pairwise analysis of building block clusters in positions p_1 and p_2

p_1 cluster id	p_2 cluster id									
	0	1	2	3	4	5	6	7	8	9
0	0.0001	0.0016	0.0000	0.0066	0.0000	0.0600	0.0001	0.0001	0.0261	0.0024
1	N/A	N/A	N/A	0.0230	N/A	0.1493	N/A	N/A	0.1469	0.0227
2	N/A	N/A	N/A	0.0264	N/A	0.0836	N/A	N/A	0.0383	0.0050
3	N/A	N/A	N/A	0.0343	N/A	0.1051	N/A	N/A	0.0595	0.0101
4	0.0000	0.0001	0.0000	0.0011	0.0000	0.0151	0.0000	0.0000	0.0090	0.0004
5	0.0003	0.0004	0.0000	0.0011	0.0000	0.0241	0.0002	0.0002	0.0186	0.0018
6	0.0001	0.0001	0.0000	0.0011	0.0002	0.0414	0.0001	0.0001	0.0327	0.0074

Table S7: Matrix entries for pairwise analysis of building block clusters in positions p_1 and p_3

p_1 cluster id	p_3 cluster id									
	0	1	2	3	4	5	6	7	8	9
0	0.0340	0.0002	0.0003	0.0002	0.0002	0.0077	0.0000	0.0002	0.0082	0.0001
1	0.1039	N/A	N/A	N/A	N/A	N/A	N/A	0.0238	0.0366	N/A
2	0.0482	N/A	N/A	N/A	N/A	N/A	N/A	0.0000	0.0088	N/A
3	0.0628	N/A	N/A	N/A	N/A	N/A	N/A	0.0048	0.0153	N/A
4	0.0106	0.0000	0.0000	0.0000	0.0000	0.0013	0.0000	0.0000	0.0002	0.0000
5	0.0190	0.0001	0.0001	0.0001	0.0000	0.0028	0.0000	0.0000	0.0000	0.0000
6	0.0291	0.0003	0.0001	0.0000	0.0003	0.0007	0.0000	0.0000	0.0138	0.0001

p_1 cluster id	p_3 cluster id								
	10	11	12	13	14	15	16	17	18
0	0.0003	0.0163	0.0185	0.0025	0.0001	0.0001	0.0250	0.0715	0.0058
1	N/A	N/A	0.1154	0.0583	0.0000	0.0000	0.0908	0.2967	0.0483
2	N/A	N/A	0.0359	0.0130	0.0000	0.0000	0.0401	0.1075	0.0065
3	N/A	N/A	0.0546	0.0229	0.0000	0.0000	0.0563	0.1630	0.0133
4	0.0000	0.0018	0.0030	0.0001	0.0000	0.0000	0.0033	0.0076	0.0000
5	0.0007	0.0031	0.0029	0.0005	0.0000	0.0001	0.0076	0.0101	0.0000
6	0.0001	0.0012	0.0196	0.0052	0.0000	0.0001	0.0176	0.0554	0.0117

Table S8: Matrix entries for pairwise analysis of building block clusters in positions p_2 and p_3

p_1 cluster id	p_3 cluster id									
	0	1	2	3	4	5	6	7	8	9
0	0.0000	0.0000	0.0001	0.0000	0.0000	0.0004	0.0000	0.0000	N/A	0.0000
1	0.3013	0.0002	0.0008	0.0009	0.0006	0.0027	0.0000	0.0000	N/A	0.0001
2	0.0000	0.0000	0.0000	0.0000	0.0000	0.0000	0.0000	0.0000	N/A	0.0000
3	0.0292	0.0006	0.0003	0.0000	0.0000	0.0378	0.0000	0.0011	0.0000	0.0000
4	0.0000	0.0000	0.0000	0.0000	0.0007	0.0002	0.0000	0.0000	N/A	0.0002
5	0.0875	N/A	N/A	N/A	N/A	N/A	N/A	0.0069	0.0216	N/A
6	0.0000	0.0001	0.0001	0.0000	0.0001	0.0000	0.0000	0.0000	N/A	0.0002
7	0.0000	0.0003	0.0001	0.0000	0.0000	0.0003	0.0000	0.0000	N/A	0.0000
8	0.0464	N/A	N/A	N/A	N/A	N/A	N/A	0.0170	0.0158	N/A
9	0.0102	N/A	N/A	N/A	N/A	N/A	N/A	0.0000	0.0010	N/A

p_1 cluster id	p_3 cluster id								
	10	11	12	13	14	15	16	17	18
0	0.0005	0.0000	0.0000	0.0002	0.0002	0.0002	0.0000	N/A	0.0000
1	0.0001	0.0089	0.0000	0.0000	0.0001	0.0003	0.0000	N/A	0.0000
2	0.0000	0.0000	0.0000	0.0000	0.0000	0.0000	0.0000	N/A	0.0000
3	0.0013	0.0696	0.0064	0.0000	0.0000	0.0000	0.0082	0.0094	0.0000
4	0.0000	0.0000	0.0000	0.0000	0.0000	0.0000	0.0000	N/A	0.0000
5	N/A	N/A	0.0731	0.0300	0.0000	0.0000	0.0736	0.2044	0.0147
6	0.0000	0.0005	0.0001	0.0005	0.0000	0.0000	0.0027	N/A	0.0000
7	0.0002	0.0014	0.0000	0.0000	0.0000	0.0000	0.0000	N/A	0.0000
8	N/A	N/A	0.0538	0.0241	0.0000	0.0000	0.0514	0.1503	0.0220
9	N/A	N/A	0.0030	0.0010	0.0000	0.0000	0.0013	0.0027	0.0002

S1.8 Full Width at Half Maximum Values

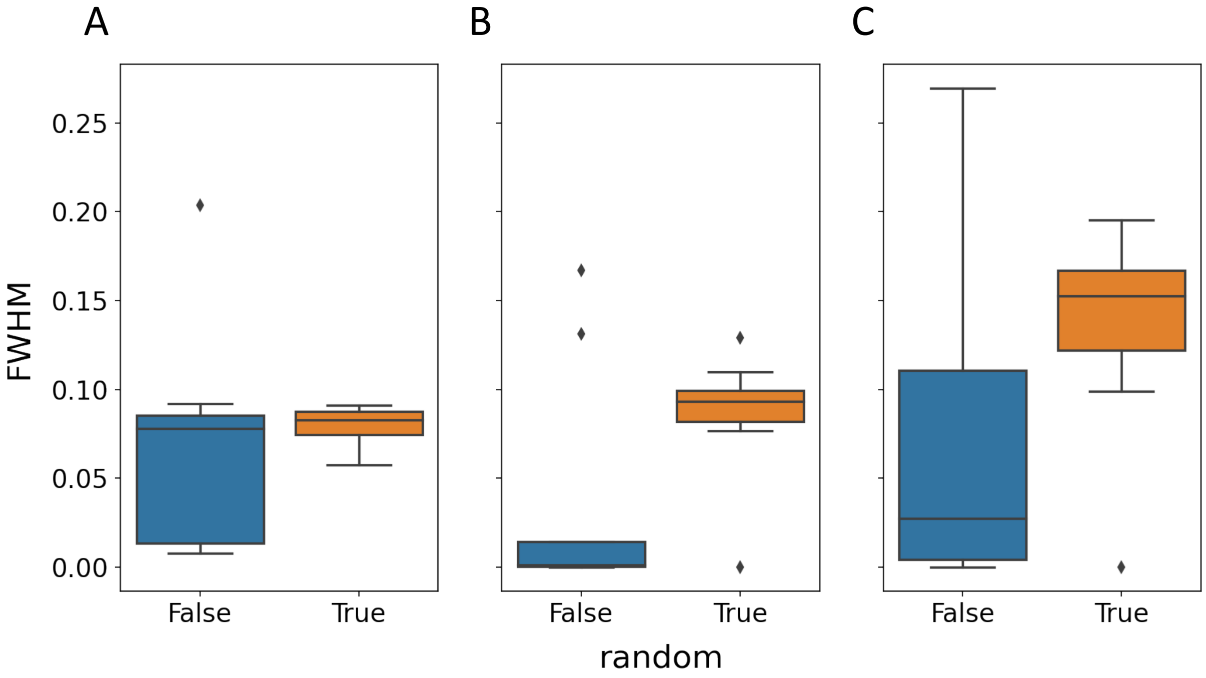

Figure S5: Boxplot of FHWM values for the distribution of P(bind) values in clusters at each building block position. The blue boxplot indicates the FWHM for the P(bind) distributions in each cluster generated using HDBSCAN. The boxplot in orange is the average FWHM of 50 different random initializations of each cluster P(bind) distribution. We observe no statistically significant difference between the FWHM of the P(bind) distributions from HDBSCAN and random clustering in (A) position 1, but do for (B) position 2 (p-value=0.0395) and (C) position 3 (p-value=1.20e-3).

S1.9 Area Under the Curve Across Multiple Random Trials

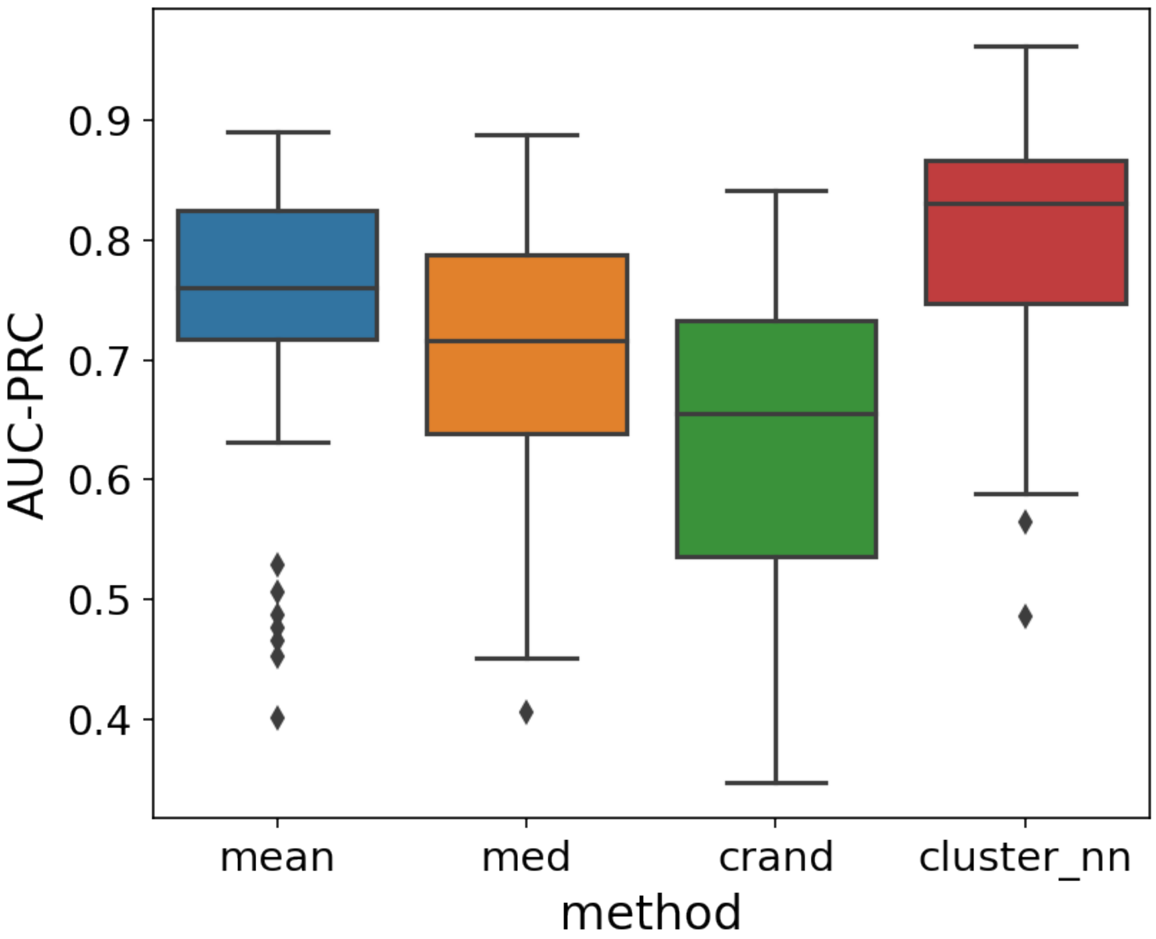

Figure S6: Performance of different prediction methods across 50 random trials.

S1.10 Diversity of Holdout Set Compound Predictions

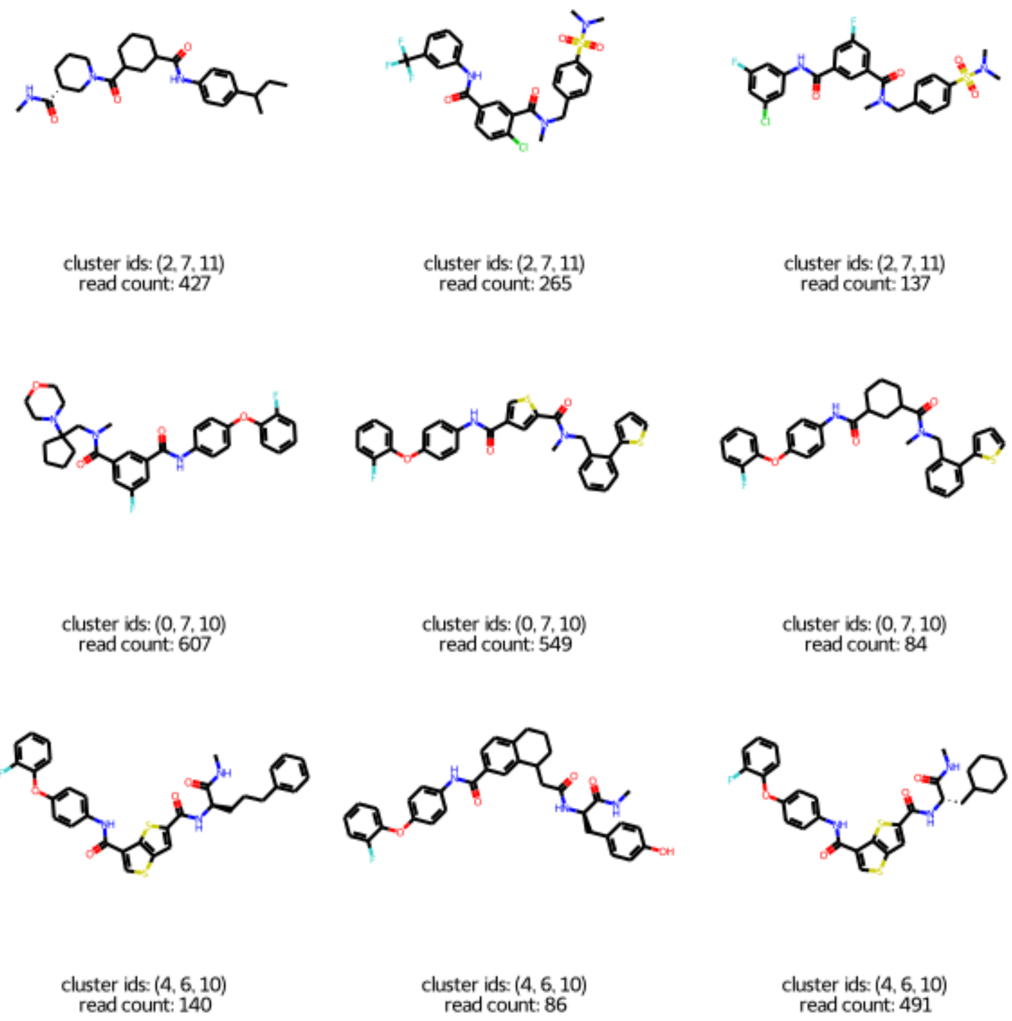

Figure S7: Examples of holdout set trisynthons predicted to bind to sEH. We trained a decision tree model to predict whether compounds containing at least one out-of-sample building block would bind to sEH. Cluster ids are given as a triplet of values, corresponding to the cluster ID for each position in the library. We did not provide read count values when training our model, but show them here to demonstrate that all the following model predictions are correct.

S1.11 Distribution of Read Count Values for Binders

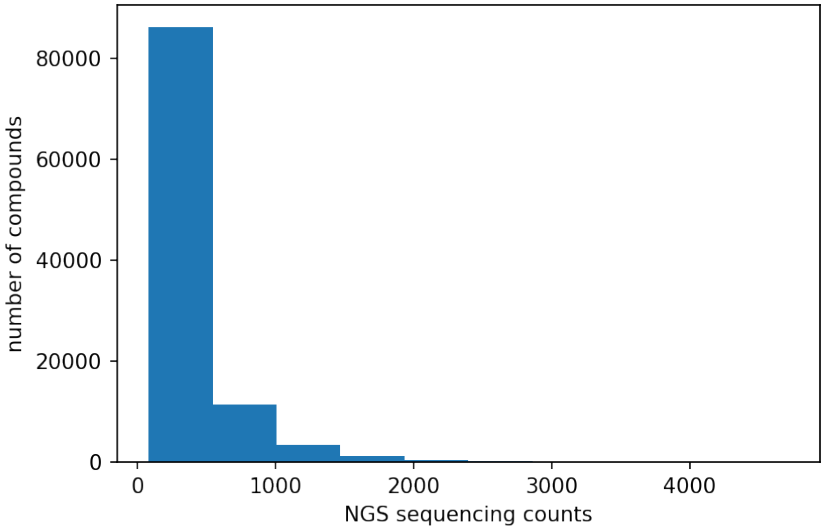

Figure S8: Distribution of NGS sequencing counts (read counts) for compounds designated as binders. From a complete set of DEL selection data screened against sEH, we selected the top 10K compounds based on read count to analyze, giving us a set of highly enriched binders. The minimum read count in our set of binders is 81 and the maximum is 4712.

Table S9: Fraction of compounds classified as binders and non-binders with varying read count threshold

Read count threshold	Fraction of binders	Fraction of non-binders
1	0.0233	0.9767
50	0.0233	0.9767
100	0.0198	0.9802
500	0.0043	0.9957
1000	0.0013	0.9987

S2 Additional Methods

S2.1 HDBSCAN Objective Function

We devised an empirical objective function to evaluate the performance of the HDBSCAN algorithm. We tried using three different metrics to evaluate clustering quality – silhouette score,[3] calinski-harabasz score[4] and davies-bouldin score[5] – and found that for every metric, there was either not enough cluster resolution, too many noise points, or too many small clusters formed (Figure S9). Thus, we created our own objective function that would score any HDBSCAN run by the number of noise (unclustered) points and the average intracluster distance. The goal was to find a set of hyperparameters that would generate compact groups of highly similar compounds, but not overfit such that many points would be left unclassified. We arrived at an empirical formula for the objective function, L

$$L = n_{noise} + 10 * ICD \tag{1}$$

We found that there was a global minimum value for the objective function across the sampled hyperparameters, meaning we could use those values as the best initialization for each HDBSCAN run (Figure S10). We selected the set of HDBSCAN parameters which would minimize the objective function.

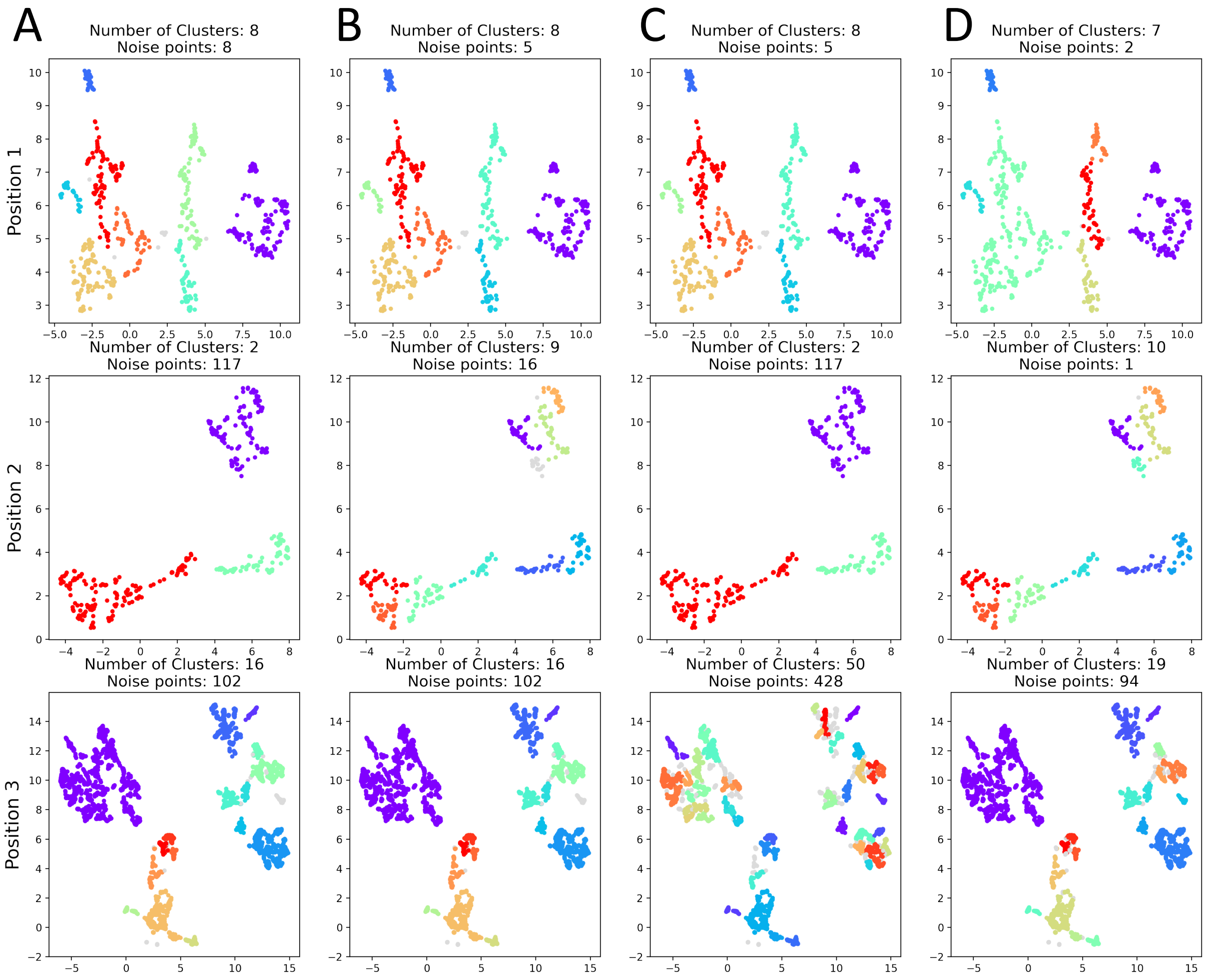

Figure S9: Cluster results for UMAP projections using different metrics. We compare clustering results when optimizing via various metrics. From left to right, (A) silhouette score, (B) calinski-harabasz score, (C) davies-bouldin score and (D) our empirical objective function. Our objective function results in the greatest number of building blocks clustered (fewest noise points) for each building block position.

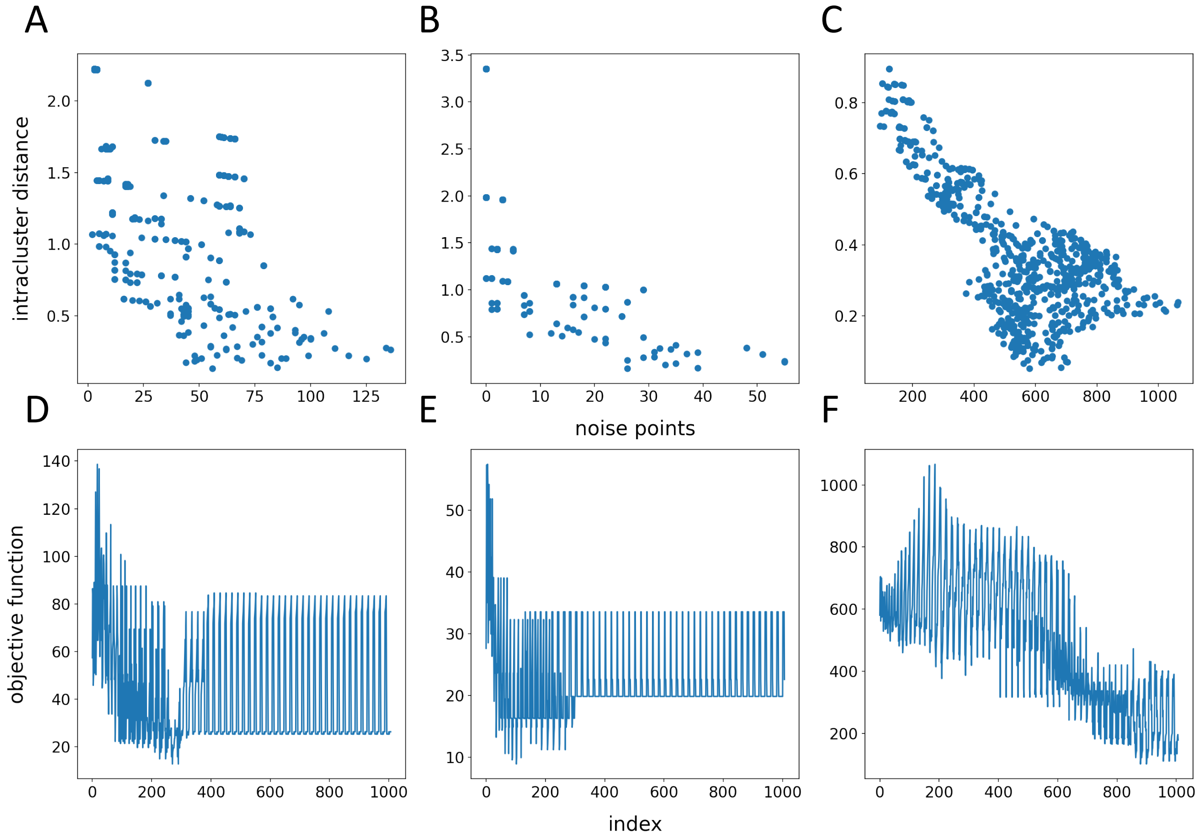

Figure S10: Relationship between the number of noise points and intracluster distance for HDBSCAN initializations. (A–C) For a given HDBSCAN initialization, we count the number of noise points and calculate the average distance between points within a cluster (intracluster distance). There is no discernable correlation between the number of noise points and intracluster distance for (A) p_1 (B) p_2 or (C) p_3. (D–F) We construct an objective function which accounts for both the number of noise points and the intracluster distance of a cluster. The index variable on the x-axis represents a distinct set of hyperparameters for HDBSCAN. We observe that the objective function has a global minimum for (D) p_1 (E) p_2 and (F) p_3. For each position, we take the set of hyperparameters corresponding to the global minimum to be the optimal set of parameters for that position.

S2.2 Selecting Decision Tree Parameter

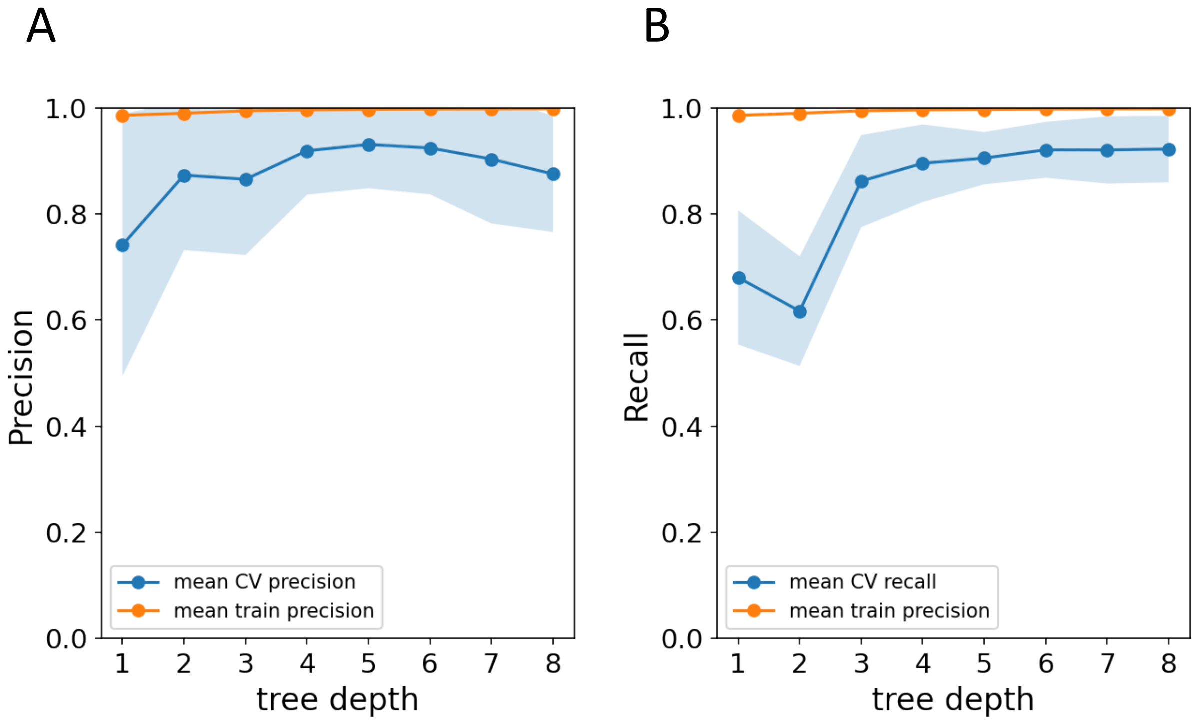

Figure S11: Hyperparameter selection for the decision tree model. We used 5-fold cross validation to find the best choice for the depth of the decision tree. For each value of tree depth, we trained a model on 4/5ths of the training data and evaluated the precision and recall of the model on the remaining 1/5th. This process was repeated 5 times to include all training points once in the validation set. We report the mean and standard deviation of both (A) precision and (B) recall across the 5 cross validation folds for each value of tree depth. We selected depth=5 as our optimal tree depth – beyond this point, the model appears to overfit, indicated by the gradual drop in model precision.

References

(1) Kwak, S. G.; Kim, J. H. Central Limit Theorem: The Cornerstone of Modern Statistics. *Korean J. Anesthesiol.* **2017**, *70*, 144–156.

(2) Eldrup, A. B.; Soleymanzadeh, F.; Taylor, S. J.; Muegge, I.; Farrow, N. A.; Joseph, D.; McKellop, K.; Man, C. C.; Kukulka, A.; De Lombaert, S. Structure-Based Optimization of Arylamides as Inhibitors of Soluble Epoxide Hydrolase. *J. Med. Chem.* **2009**, *52*, 5880–5895.

(3) Rousseeuw, P. J. Silhouettes: A Graphical Aid to the Interpretation and Validation of Cluster Analysis. *J. Comput. Appl. Math.* **1987**, *20*, 53–65.

(4) Caliński, T.; Harabasz, J. A Dendrite Method for Cluster Analysis. *Commun. Stat.* **1974**, *3*, 1–27.

(5) Davies, D. L.; Bouldin, D. W. A Cluster Separation Measure. *IEEE Trans. Pattern Anal. Mach. Intell.* **1979**, *PAMI-1*, 224–227.
